# Raloxifene Modulates Microglia and Rescues Visual Deficits and Pathology After Impact Traumatic Brain Injury

**DOI:** 10.3389/fnins.2021.701317

**Published:** 2021-10-29

**Authors:** Marcia G. Honig, Nobel A. Del Mar, Desmond L. Henderson, Dylan O’Neal, John B. Doty, Rachel Cox, Chunyan Li, Aaron M. Perry, Bob M. Moore, Anton Reiner

**Affiliations:** ^1^ Department of Anatomy and Neurobiology, The University of Tennessee Health Science Center, Memphis, TN, United States; ^2^ Department of Pharmaceutical Sciences, The University of Tennessee Health Science Center, Memphis, TN, United States; ^3^ Department of Ophthalmology, The University of Tennessee Health Science Center, Memphis, TN, United States

**Keywords:** traumatic brain injury, visual deficits, microglia, CB2 receptors, neuroinflammation, inflammatory responses, raloxifene, mouse model

## Abstract

Mild traumatic brain injury (TBI) involves widespread axonal injury and activation of microglia, which initiates secondary processes that worsen the TBI outcome. The upregulation of cannabinoid type-2 receptors (CB2) when microglia become activated allows CB2-binding drugs to selectively target microglia. CB2 inverse agonists modulate activated microglia by shifting them away from the harmful pro-inflammatory M1 state toward the helpful reparative M2 state and thus can stem secondary injury cascades. We previously found that treatment with the CB2 inverse agonist SMM-189 after mild TBI in mice produced by focal cranial blast rescues visual deficits and the optic nerve axon loss that would otherwise result. We have further shown that raloxifene, which is Food and Drug Administration (FDA)-approved as an estrogen receptor modulator to treat osteoporosis, but also possesses CB2 inverse agonism, yields similar benefit in this TBI model through its modulation of microglia. As many different traumatic events produce TBI in humans, it is widely acknowledged that diverse animal models must be used in evaluating possible therapies. Here we examine the consequences of TBI created by blunt impact to the mouse head for visual function and associated pathologies and assess raloxifene benefit. We found that mice subjected to impact TBI exhibited decreases in contrast sensitivity and the B-wave of the electroretinogram, increases in light aversion and resting pupil diameter, and optic nerve axon loss, which were rescued by daily injection of raloxifene at 5 or 10 mg/ml for 2 weeks. Raloxifene treatment was associated with reduced M1 activation and/or enhanced M2 activation in retina, optic nerve, and optic tract after impact TBI. Our results suggest that the higher raloxifene dose, in particular, may be therapeutic for the optic nerve by enhancing the phagocytosis of axonal debris that would otherwise promote inflammation, thereby salvaging less damaged axons. Our current work, together with our prior studies, shows that microglial activation drives secondary injury processes after both impact and cranial blast TBI and raloxifene mitigates microglial activation and visual system injury in both cases. The results thus provide a strong basis for phase 2 human clinical trials evaluating raloxifene as a TBI therapy.

## Introduction

Traumatic brain injury (TBI) occurs frequently as a consequence of falls, motor vehicle accidents, and sports activities, as well as with exposure to explosions during combat or training for members of the military. The vast majority of TBIs are considered to be mild, but even mild TBI, commonly known as concussion, often produces adverse sensory, motor, cognitive, and emotional outcomes. For many individuals, symptoms resolve within a few weeks (Chadehumbe, 2016), but others experience persistent problems (Hiploylee et al., 2017; Merezhinskaya et al., 2019). Among these are visual deficits, notably accommodative dysfunction, convergence insufficiency, pupil light reflex impairment, reduced visual acuity and contrast sensitivity, diminished visual fields, and photosensitivity (i.e., light aversion) (Cockerham et al., 2009; Capó-Aponte et al., 2013, 2017; Goodrich et al., 2013; Jacobs and Van Stavern, 2013; Armstrong, 2018; Fox et al., 2019; Merezhinskaya et al., 2019; Gilmore et al., 2020). For individuals whose visual deficits persist, eyeglasses with specially prescribed tints and prism combinations and oculomotor rehabilitation are currently the best solutions (Armstrong, 2018; Fox et al., 2019), as effective pharmacological treatments are lacking.

Concussive events, whether produced by an impact to the head, sudden deceleration, or an explosive blast, initiate pressure waves that are transmitted across the skull and traverse the brain parenchyma. Intracranial pressure waves then alternately compress and stretch the soft brain tissue. As axons are especially vulnerable to deformation, central visual pathways and the optic and oculomotor nerves are frequently injured in people who have experienced mild TBI (Lachapelle et al., 2004; Bruce et al., 2006; Caeyenberghs et al., 2010; Goodrich et al., 2013; Jacobs and Van Stavern, 2013). Damaged axons and their myelin sheaths release molecules, such as ATP and S100b and commonly referred to as damage-associated molecular patterns (DAMPs), which bind to receptors on microglia. Microglia in healthy adult brains possess thin, long ramifying processes that are actively motile and used to survey the local environment. In response to binding DAMPs, microglia rapidly change their morphology and transcriptional profiles and are then referred to as being “activated” or “reactive” (for example, see reviews by Hanisch and Kettenmann, 2007; Jurga et al., 2020). The initial microglial responses after TBI are typically pro-inflammatory and, as such, cause further damage and exacerbate the outcome (Loane and Kumar, 2016; Donat et al., 2017; Komorowska-Müller and Schmöle, 2021; Mesquida-Veny et al., 2021). Microglia normally express low levels of cannabinoid type-2 receptors (CB2), but rapidly upregulate CB2 levels when they become activated (Ashton and Glass, 2007; Stella, 2010; Schomberg and Olson, 2012; Donat et al., 2014; Komorowska-Müller and Schmöle, 2021), providing a means by which drugs binding CB2 receptors can selectively target microglia (Stella, 2010). While some investigators have reported benefit using CB2 agonists (Magid et al., 2019), CB2 inverse agonists show particular therapeutic promise (Lunn et al., 2006, 2008). CB2 inverse agonists stabilize the normally constitutively active CB2 receptors in an inactive state, which initiates signaling that eventually results in the phosphorylation of the cAMP response element binding protein (CREB) (Atwood et al., 2012). In turn, the transcriptional activity of phosphorylated-CREB has the overall effect of modulating microglia away from the pro-inflammatory M1 state toward the protective M2 state and thereby yields benefit (Lunn et al., 2006, 2008; Presley et al., 2015).

We have previously shown that daily treatment of mice with the selective CB2 inverse agonist SMM-189 for 2 weeks following focal cranial blast TBI greatly attenuates motor deficits, the increased depression and fear typically present at 1–2 months (Reiner et al., 2015), electrophysiological abnormalities (Liu et al., 2017), and neuron loss (Bu et al., 2016). SMM-189 treatment also rescued the contrast sensitivity deficits, optic nerve axon loss, and retinal pathologies the mice otherwise exhibit after TBI (Guley et al., 2019), with the benefit for visual system injury associated with biasing microglia toward the M2 state, as reflected in expression of the characteristic M2 state marker CD206 (Guley et al., 2019). As SMM-189 has not been tested for human use, we have more recently turned our attention to determining if raloxifene provides similar benefit. Raloxifene is approved by the Food and Drug Administration (FDA) due to its action as a selective estrogen receptor modulator, but also acts as a CB2 inverse agonist (Kumar and Song, 2013; Honig et al., 2019). If shown to be effective in animal models of TBI, raloxifene could be fast-tracked for repurposing as a TBI therapy.

We have previously reported that, similar to SMM-189, raloxifene rescues visual deficits and the associated pathologies after focal cranial blast *via* its CB2 inverse agonism (Honig et al., 2019). As emphasized in several papers addressing therapy development for TBI, because a variety of traumatic events involving different pathophysiological processes produce brain injury in the human population, researchers need to use a range of animal models to develop effective therapies (Marklund and Hillered, 2011; DeWitt et al., 2018; Hajiaghamemar et al., 2019). In particular, blast pressure waves and blunt impact generate different biomechanical forces and thus potentially may differ in the types of therapies required. Here we used a single impact to the dorsal surface of the mouse head to produce mild TBI and characterized the resulting visual deficits and pathologies. We indeed found that impact and focal cranial blast TBI exhibited important differences in their functional and pathological outcomes. Nonetheless, microglial activation worsened the outcome and raloxifene was similarly beneficial in reducing visual deficits and damage and effective in modulating microglia away from the pro-inflammatory M1 state, toward the protective M2 state. Thus, the current evidence for raloxifene benefit after impact TBI is an important step in the process of seeking regulatory approval for use in humans.

## Materials and Methods

### Animals, Traumatic Brain Injury, and Drugs

#### Animals

Male C57BL/6 mice (The Jackson Laboratory, Bar Harbor, ME, United States) received either impact or sham impact at ∼3 months of age, and they were then injected over the next 2 weeks with either raloxifene or vehicle. Visual testing began 2 months after injury and was completed at ∼8 months. Mice were then perfused transcardially with fixative, and the eyes, optic nerves, and brains were dissected and prepared for histology. Additional cohorts of 30 and 32 mice were sacrificed at 3 days after impact for immunohistochemical and molecular analysis, respectively. All experiments were performed in compliance with the Association for Research in Vision and Ophthalmology statement on the Use of Animals in Ophthalmic and Vision Research, with National Institutes of Health (NIH), Department of Defense (DOD), and The University of Tennessee Health Science Center (UTHSC) institutional guidelines, and with UTHSC institutional and DOD approval.

#### Impact Device and Administration

We used the impact model of mild TBI initially developed by Fiona Crawford and colleagues (Mouzon et al., 2012), with slight modifications. For anesthesia, mice were injected intraperitoneally (ip) with avertin (400 mg/kg). The head was shaved and the mouse was placed on a heating pad to maintain body temperature, and transferred to a stereotaxic frame (Just For Mice Stereotaxic, Stoelting, Wood Dale, IL, United States) mounted with an impact device (Impact One Stereotaxic Impactor, Leica Biosystems, Buffalo Grove, IL, United States). Ear bars with non-invasive rubber ear pad tips were pressed into the external auditory meatus, and the upper jaw incisors were placed over a bite bar, whose height was adjusted to level the skull. A 3-mm-diameter blunt metal impactor tip was retracted and positioned above the sagittal suture before each impact, centered at 1.5 mm caudal to Bregma. The injury was triggered electromagnetically and the impact was delivered at a strike velocity of 5 m/s, a strike depth of 1.0 mm, and a dwell time of 200 ms; these parameters were chosen based on experiments from the Crawford lab (Mouzon et al., 2012) showing that they produce TBI but do not fracture the skull. Mice that received a sham impact were handled the same way, except for the impact *per se*. Mice were kept warm after the impact procedure and recovered from anesthesia in 15–30 min. Acetaminophen was added to the drinking water at a final concentration of 1.6 mg/ml for 24 h before and after impact.

#### Raloxifene and Vehicle Administration

Raloxifene was diluted in vehicle containing ethanol:Cremophor:0.9% saline (5:5:90) and administered at 5 or 10 mg/kg of body weight. Mice were injected intraperitoneally with either raloxifene or vehicle, beginning at 2 h after impact, and at approximately the same time (±1 h) on subsequent days. The mice used for short-term studies received a total of four injections and the mice used for functional testing received a total of 15 doses. As described for SMM-189 in Reiner et al. (2015), the doses chosen are based on uptake into rodent brain of structurally similar CB2 compounds and drug affinity for the CB2 receptor, as well as on raloxifene efficacy as a CB2 inverse agonist in a cell culture-based assay (Honig et al., 2019). The treatment regimen is as in our prior studies demonstrating the benefit of raloxifene (Honig et al., 2019) and of the CB2 inverse agonist SMM-189 (Reiner et al., 2015; Guley et al., 2019) after focal cranial blast TBI. For brevity, mice will henceforth be referred to as impact-vehicle, sham-vehicle or simply sham, impact-ral5 for mice treated with the lower dose, and impact-ral10 for mice treated with the higher dose. One potential shortcoming of our study is that we did not include a sham-raloxifene group. We omitted this control group for two reasons. First, institutional and federal guidelines mandate minimizing the number of animals used. Second, and more importantly, microglia in sham mice express only low levels of CB2 receptors and thus would not bind raloxifene to any significant extent. Consistent with this scenario, we previously found that treatment with the CB2 inverse agonist SMM-189 did not significantly alter contrast sensitivity, motor behavior, or depression in sham mice (unpublished studies). Thus, it did not seem to us that a sham-raloxifene group would be informative in examining whether raloxifene mitigates TBI damage and deficits.

### Functional Testing

#### Contrast Sensitivity and Visual Acuity

The optokinetic reflex was used to measure contrast sensitivity thresholds and visual acuity (Cerebral Mechanics OptoMotry system; CerebralMechanics Inc.)^1^. Tests were administered in a blinded manner; the experimenter was unaware of the stimulus being presented and the eye being tested at any given time, and the treatment group. Contrast sensitivity threshold was assessed at a 0.042 c/d spatial frequency and visual acuity at 100% contrast, as in our prior studies (Reiner et al., 2015; Guley et al., 2016, 2019; Honig et al., 2019). The mice were tested before injury to ensure that there were no significant differences between experimental groups, and the results obtained after injury were normalized relative to the sham pre-injury performance.

#### Electroretinogram

Electroretinogram (ERG)s provide a non-invasive means of assessing the health of the retina in both animals and people (Cuenca et al., 2014). As rod photoreceptors constitute ∼97% of the photoreceptors in mice (Jeon et al., 1998) and the ERG responses to light flashes in dark-adapted animals are driven by rod photoreceptors, we performed scotopic ERGs. We measured the peak amplitudes of the A-wave and B-wave, following the same procedures as in our prior studies (Guley et al., 2016, 2019; Honig et al., 2019). In brief, the mice were dark adapted overnight. They were then anesthetized with ketamine-xylazine, pupils were dilated with 1% cyclopentolate hydrochloride, and methylcellulose solution (Celluvisc; Allergan, Irvine, CA, United States) was applied to each eye to protect it and maintain a good electrical connection. Gold wires on the eye served as corneal electrodes, a steel subdermal needle served as the reference electrode, and a steel needle in the tail served as the grounding electrode. The Diagnosys LLC system was used to record ERGs and to deliver a series of dim flashes to assess the peak amplitudes of the scotopic A-wave response (generated by rod photoreceptors) and the scotopic B-wave response (generated by rod bipolar cells) as follows: (1) 0.0001 cd.s/m^2^ (15 trials with a 5-s interstimulus interval, ISI); (2) 0.001 cd.s/m^2^ (10 trials with a 5-s ISI); (3) 0.01 cd.s/m^2^ (3 trials with a 10.1-s ISI); (4) 0.1 cd.s/m^2^ (3 trials with a 15.1-s ISI); (5) 1.0 cd.s/m^2^ (2 trials with a 20-s ISI); (6) 10 cd.s/m^2^ (2 trials with a 20-s ISI); and (7) 758 cd.s/m^2^ (2 trials with a 20-s ISI). ERGs were recorded before injury to ensure that there were no significant differences between experimental groups, and the results obtained after injury were normalized relative to the sham pre-injury performance.

#### Light/Dark Testing to Assess Light Aversion

Light aversion was tested in a light–dark arena using the same approach as previously described (Honig et al., 2019). In brief, the arena consisted of two equally sized compartments—an open chamber with clear Plexiglas walls and an enclosed chamber with black Plexiglas walls—with an opening allowing the mice to freely pass between the chambers. As mice are nocturnal animals, they normally spend more time in the enclosed dark chamber. The entire arena was covered by a black drape to prevent the mouse from being distracted during testing. The enclosed chamber contained a light bulb that could provide illumination at 500, 1000, or 1500 lux. The illumination of the open chamber was slightly greater than 0 lux when the arena was draped and rose to 2–10 lux with increasing illumination of the enclosed chamber. To reduce the anxiety mice exhibit when first exposed to a novel environment, they were habituated to the test arena at ∼100 days after impact, and tested about a month later (Matynia et al., 2012). Each test started with 5 min of no light in the enclosed chamber, and was followed by 5 min each of 500, 1000, and 1500 lux. As mice are sensitive to intense light, they spend less time in the enclosed chamber as it becomes brighter. Increased avoidance of the enclosed chamber, relative to sham animals, was interpreted as increased light aversion. Infrared laser beams tracked mouse movement and location.

#### Pupillometry

Pupillometry was performed on awake mice that been previously habituated by extensive handling, so they could be held with minimal manual restraint. We used a Melan-100 instrument (BioMed Vision Technologies, Ames, IA, United States), equipped with two diode-based light sources: 630 nm for red light (200 kilocandela per square meter, kcd/m^2^) and 480 nm for blue light (200 kcd/m^2^) to illuminate the eye at a distance of 4 cm, as previously described (Honig et al., 2019). Under the scotopic conditions we used for pupillometry, red light elicits pupil constriction mediated by rods, and blue light elicits constriction mediated by rods and intrinsically photosensitive retinal ganglion cells (ipRGCs), with the ipRGC response persisting for >10 s after the blue light is turned off. An image of the pupil was first taken in the dark, to determine resting pupil size. The eye was then illuminated for 2 s with the red light, allowed 10 s in the dark for recovery, and finally illuminated with the blue light. Five minutes elapsed before the other eye was tested. A digital infrared video camera (Sony Handycam; Sony Corporation) was used to image the eyes. Pupil area before, during, and after the light stimulus was measured from individual frames using ImageJ.

### Morphological and Immunohistochemical Studies

#### Animal Sacrifice

Animals were sacrificed and perfused, and tissues were dissected and prepared for later sectioning, using the same methods as previously (Guley et al., 2019; Honig et al., 2019). In brief, mice were deeply anesthetized (Avertin; 400 mg/kg ip), 0.1 ml of heparinized saline (800 U.S.P. units/ml) was injected into the heart, and the animals were perfused transcardially with 30 ml of 0.9% NaCl in 0.1 M sodium phosphate buffer at pH 7.4 (PB), followed by 60 ml of 4% paraformaldehyde, 0.1 M lysine–0.1 M sodium periodate in 0.1 M PB at pH 7.4 (PLP). Brains were removed and placed in PLP overnight 4°C to post-fix. A pin was inserted longitudinally into the right side of each brain the following day, so that the left and right sides of the brain could later be distinguished, and brains were transferred to 20% sucrose/10% glycerol and stored at 4°C until sectioned. Eyes with optic nerve attached were removed from their socket, infused with PLP, and post-fixed for 1 h at 4°C. For mice sacrificed 3 days after impact, eyes with optic nerves attached were transferred to 0.1M PB/20% sucrose/0.01% sodium azide at 4°C for storage until sectioned with a cryostat. For mice sacrificed after functional testing, the optic nerves were separated from the eyes, and post-fixed in 4% paraformaldehyde/0.5% glutaraldehyde in 0.1 M PB, for later embedding in plastic.

#### Tissue Sectioning and Processing

Tissues were sectioned and processed using procedures similar to those described previously (Guley et al., 2019; Honig et al., 2019). In brief, fixed brains were frozen with dry ice and sectioned in the transverse plane on a sliding microtome at 35 μm, collected as 12 separate series in 0.1 M PB with 0.02% sodium azide, and stored at 4°C until processing. Two series of sections were mounted as they were sectioned and stained with cresyl violet. Immunohistochemistry was performed on free-floating brain sections by peroxidase-antiperoxidase immunolabeling or by multiple immunofluorescence. Plastic-embedded optic nerves were sectioned transversely at 1 μm and stained with 1% *p*-phenylenediamine in 50% methanol. Eyes with attached optic nerves to be used for immunolabeling were cryoprotected, embedded in optimum cutting temperature compound (OCT) with optic nerve still attached, and sectioned on a cryostat at a thickness of 20 μm through the horizontal meridian. Cryostat sections were mounted onto Superfrost^®^ /Plus microscope slides, dried on a slide warmer, and stored at −20°C until immunolabeling. Image analysis, as detailed below, was performed by individuals blind to treatment group.

#### Optic Nerve Axon Counts

We counted optic nerve axons using the same approach described in our earlier studies (Guley et al., 2019; Honig et al., 2019). In brief, a low-power image of each optic nerve was captured to measure cross-sectional area and to divide the nerve into quadrants. An image of a subfield within each quadrant near its mid-point was then captured with a 100× oil immersion objective. The image of each subfield was overlain with a 4 × 6 grid of 24 100-μm^2^ counting boxes and one box per row per grid was randomly selected for counting. Axon density for each quadrant was calculated, and the densities for the four quadrants were averaged. The total number of axons was then estimated by multiplying the average axon density by the total cross-sectional area of the section of optic nerve.

#### Optic Nerve Axons and Microglia

We used cryostat sections from a cohort of 30 mice (6 sham, 8 impact-vehicle, 8 impact-ral5, and 8 impact-ral10) sacrificed 3 days after impact to examine axonal injury after impact TBI and to assess the effect of raloxifene on microglia. Some sections were stained for SMI-32 (BioLegend #801701), which recognizes non-phosphorylated 200-kD neurofilament proteins, to detect injured axons, as in our prior study (Guley et al., 2019) and with rabbit anti-IBA1 (Wako Chemicals #019-19741) to visualize all microglia, and visualized using anti-mouse IgG1 conjugated to Alexa-594 and anti-rabbit IgG conjugated to Alexa-488, respectively. Other sections were stained for multiple immunofluorescence with rabbit anti-IBA1, rat anti-CD16/32 (Abcam #ab25235) as an M1 marker, and goat anti-CD206 (R&D Systems #AF2535) as an M2 marker, as in our previous studies (Guley et al., 2019; Honig et al., 2019). IBA1 was detected with anti-rabbit IgG conjugated to Alexa-647, CD16/32 with anti-rat IgG conjugated to Alexa-594, and CD206 with anti-goat IgG conjugated to Alexa-488, all from Invitrogen. For image capture, we used a Zeiss 710 confocal microscope, a 20×, 0.8 numerical aperture objective, a pinhole setting of 2 Airy units, and the tile capture function of the Zen software. Laser power and gain were adjusted to optimize image quality and were standardized across all images for each marker. A set of two z-stacks was taken at 2-μm intervals, used to generate a maximum intensity projection image (MIP) with the Zen software, and exported as a tiff file for later analysis. The analysis of microglial markers used an approach similar to that described in Honig et al. (2019) and, as explained in the *Results* section, focused on the part of the optic nerve just beyond the extraocular muscle cone. In brief, we used the freehand pencil tool in Adobe Photoshop to outline the region of interest, measured its area, and restricted subsequent analysis to this region. Second, we measured the background labeling intensity in three small fields of view without microglia for each channel for each image and used the channel average to adjust each channel to a background OD level of 50 (1 = black, 255 = white), using the Math function in FIJI. Third, we thresholded the IBA1 channel to a standard level, and created a mask of the IBA1 labeling above threshold for each image using the Analyze Particles function in FIJI and a minimum particle size of 50 pixels (∼8.7 μm^2^). The IBA1 mask was imported back into Photoshop, compared to the labeling in the IBA1 channel, and the freehand pencil tool was used to separate neighboring microglial cells that had been incorrectly joined in FIJI. The corrected mask was then reopened in FIJI and that software was used to measure the area covered by IBA1+ microglia, count the number of IBA1+ particles ≥ 8.7 μm^2^ in size (to select individual microglia and exclude isolated processes), and measure their aspect ratios and the optical densities for each of the three channels of those particles. Aspect ratio is calculated by dividing the diameter of the major axis of an object by the diameter of the minor axis, with objects having aspect ratios greater than 1.0 being ovoid, and those with aspect ratios of 1.0 being round. In addition, as a simple descriptor of relative microglial state, we calculated an M1/M2 ratio by dividing the optical density for CD16/32 by the optical density for CD206. Analysis and imaging processing were performed blinded.

#### Optic Tract Microglia

To analyze microglial responses to impact TBI and raloxifene in the optic tract at 3 days after TBI, brain sections at the level of the optic tract were stained for multiple immunofluorescence to visualize IBA1, CD16/32, and CD206. Images were captured by confocal microscopy, as described above for optic nerve microglia, albeit with the laser power and gain settings adjusted to optimize image quality for the optic tract. Images of the optic tract on both sides of the brain were captured using the same conditions for all the brains we analyzed. Image analysis was as described above for optic nerve microglia, except that we did not measure the aspect ratios of the IBA1+ particles in the optic tract.

#### Microglia in the Retina

To analyze microglial responses in the retina, we selected cryostat sections from a level within 0.5 mm of the horizontal meridian. The sections were immunolabeled for IBA1, in conjunction with DAPI, to allow identification of retinal layers. For each eye, the tile function of the confocal software was used to capture two >1-mm-long stretches of retina, one just to the temporal side of the optic nerve, the other just to the nasal side, and a set of 5 z-stacks, taken at 2-μm intervals, was used to generate a MIP image for later image analysis.

### qPCR and NanoString Studies

#### qPCR Studies

To assess how microglia in the optic nerves, retina, and thalamus are modulated by impact TBI and raloxifene treatment, we used qPCR and NanoString Technology approaches to measure the expression of markers informative for microglial M0, M1, and M2 states. The retina and optic nerves were examined because of their obvious importance for visual function, and the thalamus because it contains the continuation of the optic nerve (i.e., the optic tract), and some of its major central targets, notably the dorsal and ventral lateral geniculate nuclei). Three days after impact, left and right optic nerves, retina, and thalamus were each separately dissected from each mouse, but because impact TBI is delivered to the dorsal midline and thus affects the two sides of the brain and the two eyes equally, tissue from the two sides was pooled together. Tissue was homogenized in TRIzol Reagent (Thermo Fisher Scientific, Waltham, MA, United States) following the manufacturer’s recommendations. RNA was isolated using the same method as in our earlier study (Honig et al., 2019). In brief, chloroform was added to the homogenate, the upper colorless phase was removed, and the lower organic phase was precipitated with 100% isopropanol and linear acrylamide. The precipitate was washed with 75% ethanol and the final mRNA pellet was suspended in diethylpyrocarbonate-treated water. RNA concentration was measured with a Qubit Spectrophotometer and RNA purity with a NanoDrop Spectrophotometer. For subsequent qPCR analysis, cDNA was generated by reverse transcription with the Transcriptor First Strand cDNA Synthesis Kit (Roche Applied Science, Mannheim, Germany), combining 100 ng of RNA with reaction buffer and enzyme mix, following the manufacturer’s directions. Amplification was required to produce an adequate amount of cDNA for qPCR from the optic nerve, with its limited volume of tissue. For purposes of standardization, we performed linear amplification for all tissues, using TaqMan^®^ PreAmp Master Mix (Thermo Fisher Scientific, Waltham, MA, United States). Microglial states were assessed using primers for the following transcripts: (1) M0—ionized calcium-binding adapter molecule 1 (IBA1), transmembrane protein 119 (Tmem119), purinergic receptor P2Y12 (P2ry12), and transforming growth factor-beta (TGFβ); (2) M1—Fc γ receptor II (CD32), interferon-γ (IFNγ), interleukin-1β (IL1β), tumor necrosis factor-alpha (TNFα), and inducible nitric oxide synthase (iNOS); and (3) M2—arginase-1 (Arg1), chitinase-like protein 3 (Ym1), interleukin-10 (IL10), and triggering receptor expressed on myeloid cells 2 (TREM2). TATA-box binding protein (TBP) was used as the housekeeping gene to standardize qPCR values. Primers and probes are shown in Table 1. Plates were run using Roche^®^ LightCycler 480 and data were analyzed using the Comparative C_*T*_ (ΔΔC_*T*_) Method. The markers examined by qPCR and by NanoString methodology (see below) were chosen because of their enrichment in microglia and their role in microglial behavior along the M0, M1, and M2 continuum (Bennett et al., 2016; Cooper and Khader, 2007; Delpech et al., 2015; Holtman et al., 2015; Jha et al., 2016; Morganti et al., 2016; Loane and Kumar, 2016; Madathil et al., 2018). Notably, 7 of the 13 transcripts we examined are unique to microglia (IBA1, Tmem119, P2ry12, CD16/32, IL1β, Ym1, and TREM2) (Cahoy et al., 2008; Bennett et al., 2016). The remaining six transcripts are typically considered to be microglial markers, but can also be expressed by astrocytes and/or neurons. While the relative expression levels of these six transcripts for the various cell types in the different tissue sources are not known, it is important to note that neurons are not present within the optic nerve. Although the microglia markers we examined are also macrophage markers, macrophages are unlikely to have been present in any of the tissues we analyzed, as macrophage invasion into the nervous system typically occurs only after more severe injuries (Anyaegbu et al., 2021; Saba et al., 2021).

**TABLE 1:** Primers and probes for TaqMan qPCR.

Gene name	Alternate name	Category	Primer 1	Primer 2	Probe #
Aif1	IBA1	M0	atctgccgtccaaacttga	ctaggtgggtcttgggaacc	67
P2ry12	P2ry12	M0	gtgggcgtaccctacagaaa	aggcagccttgagtgttctt	109
Tgfb1	TGFβ	M0	tggagcaacatgtggaactc	gtcagcagccggttacca	72
Tmem119	Tmem119	M0	gtcactccatcccagtttcac	ggaccatgttgagctatggaa	110
Fcgr2b	CD32	M1	tactgtggacagccgtgcta	ttgaccacagcctttggaa	3
Ifng	IFNγ	M1	atctggaggaactggcaaaa	ttcaagacttcaaagagtctgagg	21
IL1b	IL1β	M1	agttgacggaccccaaaag	agctggatgctctcatcagg	38
Tnf	TNFα	M1	gccaacatccctacctctcc	ccccagggcaaaggtaat	92
Nos2	iNOS	M1	ctttgccacggacgagac	tcattgtactctgagggctgac	13
Arg1	Arg1	M2	ggcaaggtgatggaagagac	aggtgaatcggccttttctt	3
Chil3	Ym1	M2	ggtctgaaagacaagaacactgag	gagaccatggcactgaacg	88
IL10	IL10	M2	cagagccacatgctcctaga	tgtccagctggtcctttgtt	41
Trem2	Trem2	M2	cgagaggctgaggtcctg	tctccagcatcttggtcatcta	22
Tbp	TBP	HK	ggtcgcgtcattttctcc	gggttatcttcacacaccatga	107

*Gene targets (plus common name), gene category with regard to microglial state, and primer pairs and TaqMan probes used to detect them in qPCR analysis.HK, housekeeping.*

#### NanoString Studies

Some of the mRNA we harvested was used for NanoString analysis of expression instead of RT/qPCR. With this assay, hundreds of mRNA transcripts can be detected simultaneously and their abundance measured directly from RNA, without it having to be converted into cDNA (Kulkarni, 2011). As described by the manufacturer (NanoString Technologies, Seattle, WA, United States), the technology is based on gene-specific probe pairs that hybridize directly to the target mRNA—a reporter probe that carries a fluorescent signal, and a capture probe that allows the complex to be immobilized for signal quantification. Hybridization, purification, and detection of target-probe complexes was performed with the nCounter system. nSolver 4.0 (Version 2.1.115) was used for quality control, normalization, and measurement of transcript levels. We assessed the expression of the following transcripts enriched in microglia: (1) M0—proto-oncogene tyrosine-protein kinase MER (MERTK), P2ry12, secreted protein acidic and rich in cysteine (SPARC), and Tmem119; (2) M1—Fc γ receptor IIIb (CD16), CD32, IFNγ, IL1β, interleukin-6 (IL6), iNOS, T-lymphocyte activation antigen (CD86), interleukin-12 subunit β (IL12p40), monocyte chemoattractant protein-1 (MCP-1), and toll-like receptor-2 (Tlr2); and (3) M2—Arg1, mannose receptor C type-1 (CD206), IL10, Trem2, Ym1, resistin-like β (Fizz1), platelet glycoprotein-4 (CD36), interleukin-13 (IL13), and interleukin-4 receptor α chain (IL4r-α). Note that 14 of these transcripts are negligibly expressed (if at all) by astrocytes, oligodendrocytes, or neurons (Tmem119, P2ry12, CD16, CD32, CD86, IFNγ, TLR2, CD206, CD36, Fizz1, IL13, IL4r-α, TREM2, and YM1) (Cahoy et al., 2008; Bennett et al., 2016). The housekeeping genes used as standards were ATP-binding cassette sub-family F member 1 (Abcf1), β-glucuronidase (GusB), hypoxanthine-guanine phosphoribosyltransferase (Hprt), lactate dehydrogenase A (Ldha), RNA polymerase subunit B Polr1b (Polr1b), and ribosomal protein lateral stalk subunit P0 (Rplp0). The mRNA sequence and the probe used for each gene are shown in Table 2. The relative levels of expression compared to sham for each tissue for the genes examined by both qPCR and NanoString were very similar and so, in those cases, we used the average of the two values. We also averaged the data for the M1 and M2 markers to provide an overview of microglial phenotypes and we expressed the M1 and M2 averages as a ratio to serve as a simple descriptor of the overall bias toward one state or the other.

**TABLE 2:** Gene targets and probes for NanoString analysis.

Gene name	Alternate name	Category	Accession number	mRNA region targeted	Sequence targeted	NanoString probe
Aif1	IBA1	M0	NM_019467.2	56–155	CTGGAGCAGCCTGCAGACTTCATCCTCTCTCTTCCA TCCCGGGGAAAGTCAGCCAGTCCTCCTCAGCT GCCT GTCTTAACCTGCATCATGAAGCCTGAGG	NM_019467.2:55
Mertk	Mertk	M0	NM_008587.1	1321–1420	AGGGACTTACAAAGAGCTTTCTGAAGAAGTCAGCCAGA ATGGCAGCTGGGCTCAGATTCCTGTCCAAA TCCACAATGCCACCTGCACAGTGAGAATCGCG	NM_008587.1:1320
P2ry12	P2ry12	M0	NM_027571.3	440–539	GATCACCCAGGTTCTCTTCCCATTGCTGTACACCGTCC TGTTCTTTGCTGGGCTCATCACGAACAGCTTG GCAATGAGGATTTTCTTTCAGATCCGCAGT	NM_027571.3:439
Sparc	SPARC	M0	NM_009242.4	175–274	CTCCAAGAGGCTTCCTGCTGCTCGCCTCTAAACCCCTCCA CATTCCTGCAGCCCTTCAGACCGCCAGAACTCT TCTGCCGCCTGCCTGCCTGCCTGCCTG	NM_009242.4:174
Tmem119	Tmem119	M0	NM_146162.2	1551–1650	TGGCTACTCTAAGGGTTCCTGCTGGGCTGGCT TTGCTACGCTTTCCTCAAGCTGCTTTCTTATTACCAGGA TGCCTCACAGCTACAAAGTCCAATCTCAC	NM_146162.2:1550
Cd86	CD86	M1	NM_019388.3	252–351	CAAAACATAAGCCTGAGTGAGCTGGTAGTATTTTGGCAGGA CCAGCAAAAGTTGGTTCTGTACGAGCACTATTTGGGC CAGAGAAACTTGATAGTGTGA	NM_019388.3:251
Fcgr2b	CD32	M1	NM_001077189.1	1226–1325	TTGGTTCCCAATGGTTGACTGTACTAATGACTCCCATAA CTTACAGCTTCCCAACTCAAGACTCT TCTGCTATCGATCCACACTGCCACTAAAATTAATC	NM_001077189.1:1225
Fcgr3	CD16	M1	NM_010188.5	1176–1275	TCTGACCTCCACCATCCACCATGGCAGGTGCACACAAT AAATTAAAATGTCATGTATATTTTTAAACAAGAGACAG GGGCAGGCTAAGGGTTGATGGCAT	NM_010188.5:1175
Ifng	IFNg	M1	NM_008337.1	96–195	CTAGCTCTGAGACAATGAACGCTACACACTGCATCT TGGCTTTGCAGCTCTTCCTCATGGCTGTTTCTGGCTGTTA CTGCCACGGCACAGTCATTGAAAG	NM_008337.1:95
IL12b	IL12p40	M1	NM_001303244.1	415–514	TCAAAAACAAGACTTTCCTGAAGTGTGAAGCAC CAAATTACTCCGGACGGTTCACGTGCTCATGGCTGGT GCAAAGAAACATGGACTTGAAGTTCAACAT	NM_001303244.1:414
IL13	IL13	M1	NM_008355.2	426–525	AGCTACACAAAGCAACTGTTTCGCCACGGCCCCTTCTAAT GAGGAGAGACCATCCCTGGGCATCTCAGCTGTGGACTC ATTTTCCTTTCTCACATCAGAC	NM_008355.2:425
IL1b	IL1b	M1	NM_008361.3	1121–1220	GTTGATTCAAGGGGACATTAGGCAGCACTCTCTAGAAC AGAACCTAGCTGTCAACGTGTGGGGGATGAATTGGTCA TAGCCCGCACTGAGGTCTTTCATT	NM_008361.3:1120
IL6	IL6	M1	NM_031168.1	41–140	CTCTCTGCAAGAGACTTCCATCCAGTTGCCTTCTTGGGACTG ATGCTGGTGACAACCACGGCCTTCCCTACTTCACAAGT CCGGAGAGGAGACTTCACAG	NM_031168.1:40
Mcpt1	MCP-1	M1	NM_008570.1	366–465	ATGTTACTGAAGCTTGAAGAGAAAGCTGAGTTGACTC CTACTGTGGATGTAATTCCCTTGCCTGGTCCCTCTGA CTTTATCGACCCTGGGAAGATGTGCT	NM_008570.1:365
Nos2	iNOS/NOS2	M1	NM_010927.3	3716–3815	CCCCCCTCCTCCACCCTACCAAGTAGTATTG TACTATTGTGGACTACTAAATCTCTCTCCTCTCCTCCC TCCCCTCTCTCCCTTTCCTCCCTTCTTCTCC	NM_010927.3:3715
Tlr2	Tlr2	M1	NM_011905.2	256–355	GCAGGCGGTCACTGGCAGGAGATGTGTCCGCAATCATAGTTTC TGATGGTGAAGGTTGGACGGCAGTCTCTGCGACCT AGAAGTGGAAAAGATGTCGTTC	NM_011905.2:255
Arg1	Arg1	M2	NM_007482.3	627–726	GTACATTGGCTTGCGAGACGTAGACCCTGGGGAACACTATA TAATAAAAACTCTGGGAATTAAGTATTTCTCCATGA CTGAAGTAGACAAGCTGGGGATT	NM_007482.3:626
Cd36	CD36	M2	NM_007643.3	1521–1620	GGGACCATTGGTGATGAAAAAGCAGAAATGTTCAAAACA CAAGTGACTGGGAAAATCAAGCTCCTTGGCATGGTAG AGATGGCCTTACTTGGGATTGGAG	NM_007643.3:1520
Chil3	YM1	M2	NM_009892.2	824–923	ATTGTGGGATTTCCAGCATATGGGCATACCTTTATCCTGA GTGACCCTTCTAAGACTGGAATTGGTGCCCCT ACAATTAGTACTGGCCCACCAGGAAAGT	NM_009892.2:823
IL10	IL10	M2	NM_010548.1	986–1085	GGGCCCTTTGCTATGGTGTCCTTTCAATTGCTCTCAT CCCTGAGTTCAGAGCTCCTAAGAGAGTTGTGAAGA AACTCATGGGTCTTGGGAAGAGAAACCA	NM_010548.1:985
IL4ra	IL4r-alpha	M2	NM_001008700.3	671–770	TGGAATAACCTGTACCCATCGAACAAC TTACTGTACAAAGACCTCATCTCCATGGTCAACAT CTCCAGAGAGGACAACCCTGCAGAATTCATAGTCTATA	NM_001008700.3:670
Mrc1	CD206	M2	NM_008625.1	3993–4092	GTTCCGAAATGTTGAAGGGAAGTGGCTTTGGTTG AACGACAATCCTGTCTCCTTTGTCAACTGGAAAAC AGGCGATCCCTCTGGTGAACGGAATGATTGT	NM_008625.1:3992
Retnla	Fizz1	M2	NM_020509.3	165–264	GAATACTGATGAGACCATAGAGATTATCGTGGAG AATAAGGTCAAGGAACTTCTTGCCAATCCAGCTAA CTATCCCTCCACTGTAACGAAGACTCTCTCT	NM_020509.3:164
Trem2	Trem2	M2	NM_031254.2	8–107	GGGCGCCTACCCTAGTCCTGACTGTTGCTCAATCCA GGAGCACAGTTCCTGTGGGCTGAGCCTGACT GGCTTGGTCATCTCTTTTCTGCACTTCAAGGGA	NM_031254.2:7
Abcf1	Abcf1	HK	NM_013854.1	876–975	GAGGTGTCTTCCCGCCAGGCAATGTTAGAAAA TGCATCTGACATTAAGTTGGAAAAGTTCAGCATCTCCGCCCA CGGCAAGGAGCTATTCGTCAATGCTG	NM_013854.1:875
Gusb	Gusb	HK	NM_010368.1	284–383	CCCTTCGGGACTTTATTGGCTGGGTGTGGTAT GAACGGGAAGCAATCCTGCCACGGCGATGGACCCAAGA TACCGACATGAGAGTGGTGTTGAGGATCAA	NM_010368.1:283
Hprt	Hprt	HK	NM_013556.2	31–130	TGCTGAGGCGGCGAGGGAGAGCGTTGGGCTTAC CTCACTGCTTTCCGGAGCGGTAGCACCTCCTCCGCCGGCTT CCTCCTCAGACCGCTTTTTGCCGCGA	NM_013556.2:30
Ldha	Ldha	HK	NM_010699.1	256–355	CAGAACAAGATTACAGTTGTTGGGGTTGGTGCT GTTGGCATGGCTTGTGCCATCAGTATCTTAATGAAGGA CTTGGCGGATGAGCTTGCCCTTGTTGACG	NM_010699.1:255
Polr1b	Polr1b	HK	NM_009086.2	2796–2895	TGCCTTTCACTGAGAGTGGCATGATGC CGGACATTCTGTTTAATCCTCACGGGTTTCCCTCCCGTATGA CCATAGGTATGTTAATCGAGAGCATGGCTGG	NM_009086.2:2795
Rplp0	Rplp0	HK	NM_007475.5	496–595	TCAGAACACTGGTCTAGGACCCGAGAAGACCT CCTTCTTCCAGGCTTTGGGCATCACCACGAAAATCTCCAGA GGCACCATTGAAATTCTGAGTGATGTG	NM_007475.5:495

*Gene targets (plus common name), gene category with regard to microglial state, and probes used to detect them in qPCR analysis. HK, housekeeping.*

### Statistical Analysis

One-way ANOVA followed by planned comparisons using *post hoc* Fisher Protected Least Significant Difference tests was used to analyze contrast sensitivity, visual acuity, pupil responses, optic nerve axon counts, and specific microglial endpoints. ERGs and light aversion were analyzed by one-way ANOVA with planned comparisons between groups across light intensities. Chi-square was used to compare sets of microglial characteristics across experimental groups. Results are presented as group mean ± standard error of the mean (SEM).

## Results

Here we report the consequences of TBI produced by an impact to the dorsal surface of the head and the benefit provided by raloxifene for visual function and associated morphological changes. Each cohort of mice consisted of four groups: impact mice injected with vehicle, impact mice treated with 5 mg/kg raloxifene, impact mice treated with 10 mg/kg raloxifene, and sham impact mice injected with vehicle. Most mice were used for visual testing, which was carried out in the following sequence: contrast sensitivity and visual acuity ∼2 months after impact, ERGs at ∼2.5 months, light aversion at ∼5 months, and pupillometry at ∼8 months. Mice were then sacrificed for histological analyses. Two small cohorts of mice were sacrificed 3 days after impact to evaluate microglial state. Note that as the impact injury is at the dorsal midline, the eyes, optic nerves, and the two sides of the brain were equally affected. Thus, for each experimental group, we pooled the data for the two eyes, optic nerves, or sides of the brain.

### Functional Benefit of Raloxifene

#### Contrast Sensitivity and Visual Acuity

**FIGURE 1:**
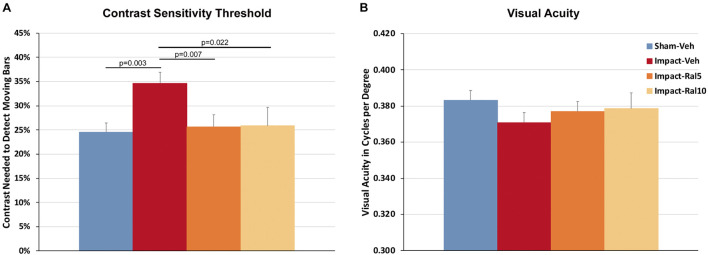
Contrast sensitivity and visual acuity as measured using Optometry ∼2 months after impact. **(A)** The contrast sensitivity thresholds for both eyes were significantly higher in impact-vehicle mice than in sham-vehicle mice. Mice treated with raloxifene were similar to sham and improved over impact-vehicle mice. **(B)** Visual acuity for impact-vehicle mice was somewhat diminished compared to sham mice, but the reduction was not statistically significant. Mice treated with raloxifene showed a smaller reduction in visual acuity than the impact-vehicle mice. Data are pooled for the two eyes. Errors bars are SEMs. Animal numbers: 21 sham-vehicle mice, 26 impact-vehicle mice, 22 impact-ral5 mice, and 14 impact-ral10 mice.

Impact TBI produced a significant deficit in contrast sensitivity with the impact-vehicle mice needing greater contrast to detect the moving stripes than the sham mice (*p* = 0.003; Figure 1A). Impact mice treated with 5 mg/kg or with 10 mg/kg raloxifene exhibited better contrast sensitivity (*p* = 0.007 and *p* = 0.022, respectively) than the impact-vehicle mice and were not significantly different from the sham mice. Thus, raloxifene rescued the deficit in contrast sensitivity produced by impact TBI. Visual acuity showed a small reduction in the impact-vehicle mice compared to the sham-vehicle mice that did not achieve significance (Figure 1B). Visual acuity for the impact mice treated with both doses of raloxifene was slightly better than for the impact-vehicle mice and not significantly different than for the sham mice, suggestive of improvement.

#### Scotopic Dark-Adapted Electroretinogram A-Wave and B-Wave

**FIGURE 2:**
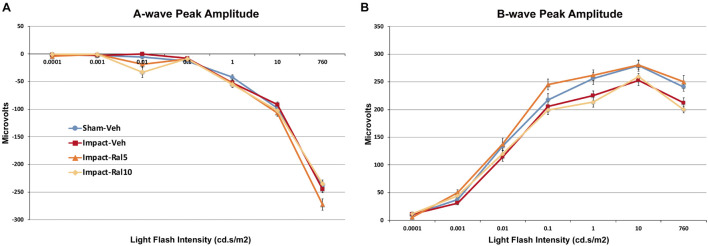
Average flash-evoked scotopic ERG peak A-wave and B-wave amplitudes at ∼2 months after impact. **(A)** A-wave peak amplitudes, compared across the three brightest light intensities, were similar for sham-vehicle, impact-vehicle, and impact-ral10 mice, but were increased for the impact-ral5 mice (*p* = 0.0006 relative to sham). **(B)** B-wave peak amplitudes, compared across the six brightest light intensities, were reduced for impact-vehicle mice (*p* = 2.8 × 10^− 5^ relative to sham). Impact mice treated with 5 mg/kg raloxifene showed rescue of the B-wave deficit, with amplitudes increased compared to impact-vehicle mice (*p* = 2.0 × 10^− 10^). Raloxifene at 10 mg/ml did not yield rescue, with B-wave amplitudes similar to impact-vehicle mice and significantly less than in sham (*p* = 3.2 × 10^− 5^). Errors bars are SEMs. Animal numbers: 15 sham-vehicle mice, 17 impact-vehicle mice, 17 impact-ral5 mice, and 14 impact-ral10 mice.

The average peak amplitudes of the A-wave responses (Figure 2A) were similar in impact-vehicle mice and sham mice at all light intensities. This was unchanged by 10 mg/kg raloxifene, but 5 mg/kg raloxifene significantly enhanced the A-wave response over the three brightest light intensities (*p* = 0.0006). By contrast, impact TBI produced a B-wave deficit (Figure 2B), with the average peak amplitudes of the B-wave over the brightest six intensities significantly reduced in impact-vehicle mice compared to sham mice (*p* = 2.8 × 10^–5^). These results indicate that, although rod photoreceptors were not harmed by the impact, rod bipolar cells were somehow impaired. Raloxifene at 5 mg/kg yielded prominent rescue, with the B-wave responses across the brightest six intensities significantly greater than in impact-vehicle mice (*p* = 2.0 × 10^–10^) and even greater than in sham mice (*p* = 0.041). Surprisingly, raloxifene at 10 mg/kg did not rescue the B-wave deficit created by impact TBI, with B-wave responses significantly smaller than in sham mice (*p* = 3.2 × 10^–5^) and similar to impact-vehicle mice.

#### Light Aversion

**FIGURE 3:**
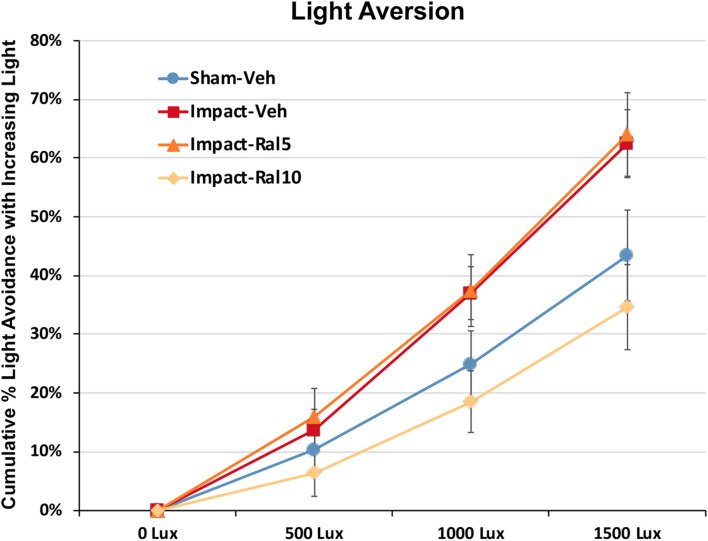
Light aversion at ∼5 months after impact. Light aversion was plotted as cumulative avoidance of an enclosed chamber with increasing brightness, relative to an adjacent dark chamber. Impact-vehicle mice and impact-ral5 mice showed greater avoidance of the enclosed chamber with increasing light intensity than sham-vehicle mice across the three levels of illumination (*p* = 0.031). Mice treated with 10 mg/kg raloxifene, however, exhibited less light aversion than impact-vehicle mice (*p* = 0.029) and impact-ral5 mice (*p* = 0.033) but not significantly differently than sham mice. Thus, 10 mg/kg raloxifene, but not 5 mg/kg, rescued the increase in light aversion that resulted from impact TBI. Errors bars are SEMs. Animal numbers: 16 sham-vehicle mice, 20 impact-vehicle mice, 17 impact-ral5 mice, and 14 impact-ral10 mice.

Light aversion was assessed by allowing the mice to choose between an open chamber kept dark and an enclosed chamber with variable illumination. As shown in Figure 3, increased illumination in the enclosed chamber resulted in its greater avoidance for all groups of mice. Both the impact-vehicle mice and the impact mice treated with 5 mg/kg raloxifene, however, exhibited significantly greater light aversion than sham mice, spending less time than sham mice in the increasingly brighter enclosed chamber (*p* = 0.031). Treatment with 10 mg/kg raloxifene rescued the heightened light aversion, with impact-ral10 mice occupying the illuminated enclosed chamber significantly less than impact-vehicle mice (*p* = 0.029) and impact-ral10 mice (*p* = 0.033). Thus, impact TBI caused increased light aversion, and the higher dose of raloxifene was needed to prevent this.

#### Pupil Light Reflex

**FIGURE 4:**
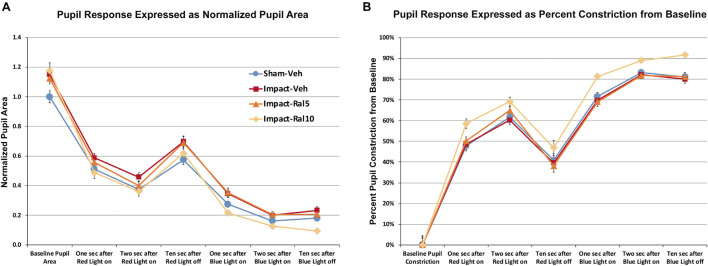
Pupil responses to red and blue light ∼7 months after impact. **(A)** Pupil area is shown normalized to the pre-illumination baseline for sham mice. Pupil size at rest was greater in all groups of impact mice (including drug treated) than in sham mice (*p* = 0.0362), as well as during red light in the case of impact-vehicle mice (*p* = 0.0059) and blue light in the case of impact-vehicle mice (*p* = 0.0009) and impact-ral5 mice (*p* = 0.0033). **(B)** Pupil size is plotted as the % constriction from baseline for each group of mice. The % constriction during red and blue light was no different in sham, impact-vehicle, and impact-ral5 mice. Although 10 mg/kg raloxifene did not rescue the increase in resting pupil size caused by impact (*p* = 0.0086), it did normalize the absolute magnitude of constriction caused by red light and yielded hyperconstriction to blue light (*p* = 0.0014). As a result, % pupil constriction from baseline in response to blue light was significantly greater in impact-ral10 mice than in sham (*p* = 7.6 × 10^− 8^), as was the % constriction to red light (*p* = 0.0001). Errors bars are SEMs. Animal numbers: 21 sham-vehicle mice, 25 impact-vehicle mice, 22 impact-ral5 mice, and 14 impact-ral10 mice.

We found that pupil area was approximately 15% greater in impact TBI-vehicle mice before red light (*p* = 0.009), during red light (*p* = 0.006), after red light (*p* = 0.022), and during and after blue light (*p* = 0.0008) compared to sham mice (Figure 4A), but the percent change in constriction from baseline was not significantly different than in sham (Figure 4B). Thus, the pupil was excessively dilated at rest and during light-evoked constriction after impact TBI, but the relative magnitudes of the dynamic constriction to red light (driven by photoreceptors) and to blue light (driven by ipRGCs) were similar to those in sham mice. Treatment with 5 mg/kg raloxifene did not rescue the enlarged resting pupil size compared to sham (*p* = 0.036) or the excessive dilation during blue light compared to sham (*p* = 0.003), although there was a slight, but not significant, rescue of the excessive dilation during red light, and the percent constriction from baseline to both red light and blue light was normal. For impact mice treated with 10 mg/kg raloxifene, resting pupil size was again significantly greater than for sham mice (*p* = 0.009), but pupil size during red light was not significantly different than in sham and was significantly less during blue light than in sham (*p* = 0.001) (Figure 4A). Thus, 10 mg/kg raloxifene did not rescue the increase in resting pupil size caused by impact TBI, but it did normalize the absolute magnitude of constriction caused by red light, and it yielded a relative hyperconstriction to blue light. Due to this differential pattern of rescue, the percent pupil constriction from baseline in response to blue light and to red light was significantly greater in impact mice treated with 10 mg/kg raloxifene than in sham mice (*p* = 7.6 × 10^–8^ and *p* = 0.0001, respectively) (Figure 4B). Thus, treatment with 10 mg/kg raloxifene rescued the pupillary defect (i.e., excessive dilation) caused by impact TBI and caused an exaggerated percent constriction from baseline.

### Morphological Benefit of Raloxifene for Optic Nerve Axon Loss

**FIGURE 5:**
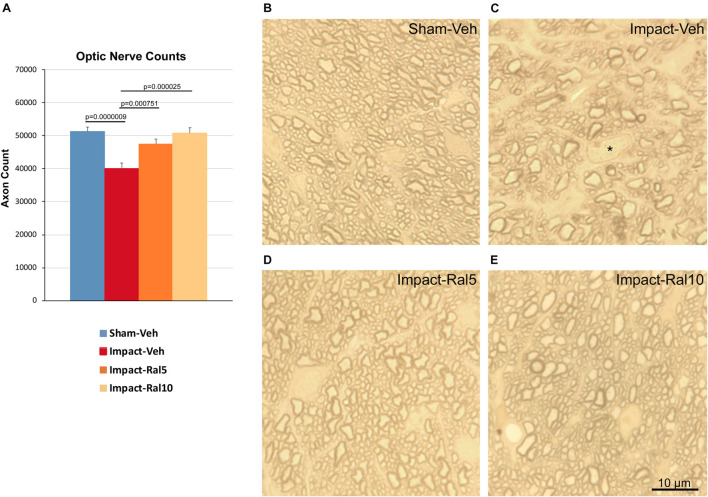
Optic nerve axon loss. **(A)** Impact-vehicle mice showed a significant 21.8% loss of optic nerve axons compared to sham mice. Treatment with 5 mg/kg raloxifene yielded partial rescue, with only a 7.4% reduction in optic nerve axons, such that axon abundance was significantly greater than for the impact-vehicle mice and only trended toward being significantly different from sham (*p* = 0.084). With raloxifene at 10 mg/kg, axon abundance was again significantly greater than in the impact-vehicle mice and indistinguishable from that in sham mice. Errors bars are SEMs. Animal numbers: 21 sham-vehicle mice, 25 impact-vehicle mice, 21 impact-ral5 mice, and 13 impact-ral10 mice. **(B–E)** High-magnification views of the optic nerve in sham-vehicle, impact-vehicle, impact-ral5, and impact-ral10 mice. The density of axons is obviously lower in the image from the impact-vehicle mouse, and glial cells occupy relatively more space than in the image from the sham mouse. The asterisk in C marks the nucleus of a glial cell. By contrast, axon density in the images from the impact-ral5 and impact-ral10 mice appears similar to that in the image from the sham mouse. Scale bar in **(E)** applies to **(B–E)**.

We found a significant 21.8% loss of optic nerve axons in the impact-vehicle mice compared to sham mice (*p* = 9.0 × 10^–7^) ∼8 months after impact TBI (Figure 5). By contrast, the impact mice treated with 5 mg/kg raloxifene showed only a 7.4% reduction of optic nerve axons, which was significantly greater than for the impact-vehicle mice (*p* = 0.0008) and trended toward being significantly different from sham (*p* = 0.084). Raloxifene at 10 mg/kg was more effective for axon rescue, with an axon abundance that was statistically indistinguishable from that in sham mice and significantly greater than in the impact-vehicle mice (*p* = 2.5 × 10^–5^). Optic nerve axon abundance did not correlate with either contrast sensitivity threshold or visual acuity across all groups of animals.

### Microglial Modulation by Raloxifene

An impact to the dorsum of the head is known to damage axons of retinal origin (Bolton-Hall et al., 2016; Tzekov et al., 2014, 2016a, b). Following focal cranial blast TBI (Guley et al., 2019; Honig et al., 2019), we have previously shown such damage in the form of swollen axon bulbs and activated microglia in the optic nerve and optic tract during the first few days after injury. Here we examined mice using immunohistochemical and molecular approaches at the same 3-day time point as in our previous studies.

#### Effect on Optic Nerve Axons and Microglia

**FIGURE 6:**
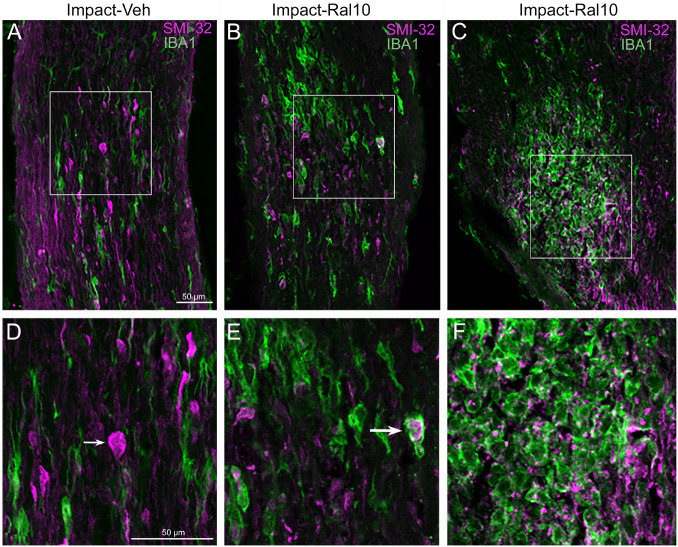
Axon injury and microglial activation in the optic nerve. Confocal images of optic nerve sections from mice 3 days after impact TBI, taken from the region just beyond the extraocular muscle cone, immunostained with SMI-32 to detect injured axons and for IBA1 to reveal microglia. **(A)** Large SMI-32+ axon bulbs (magenta) and intensely stained microglia (green), indicative of injury to the nerve, are visible in the image from an impact-vehicle mouse. Note that, by contrast, in sham mice, axon bulb pathology is absent (and thus not shown here) and microglia are lightly labeled for IBA1 (see Figure 7A). **(B)** Microglia with more rounded shapes are present in impact mice treated with 10 mg/kg raloxifene, one of which appears to be engulfing an axon bulb. **(C)** Numerous microglia are seen clustered together, amidst a field of small SMI-32+ profiles that resemble debris from degenerating axons. **(D–F)** Higher-magnification views of the regions shown boxed in **(C)**. The small arrow in **(D)** marks an especially large axon bulb. The larger arrow in **(E)** indicates a rounded microglial cell that appears to be engulfing an axon bulb. Scale bar in **(A)** applies to **(A–C)**, and scale bar in **(D)** applies to **(D–F)**.

To assess optic nerve damage, we began by combining immunolabeling for IBA1, to visualize microglia, and immunostaining with the SMI-32 monoclonal antibody, which detects non-phosphorylated 200-kD neurofilament proteins, to visualize damaged axons. As with focal cranial blast TBI (Guley et al., 2019), we found swollen axon bulbs at a level slightly beyond the extraocular muscle cone and thus within, or slightly past, the bony optic canal (Figures 6A,D). Microglia in this region typically were intensely immunostained for IBA1 and possessed large cell bodies, both signs characteristic of microglial responses to axon damage (Soltys et al., 2001; Davis et al., 2017). By contrast, we observed few, if any, axon bulbs or activated microglia at either the head of the optic nerve or along its intraorbital course.

**FIGURE 7:**
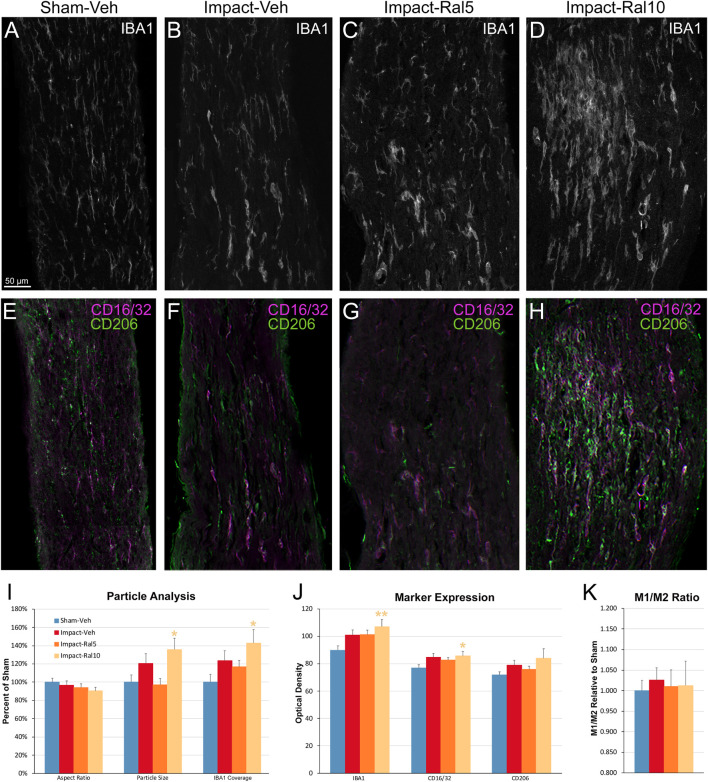
Microglia in the optic nerve 3 days after impact TBI. **(A–F)** Confocal images of sections immunostained for IBA1 to visualize microglia, for the M1 marker, CD16/32, and for the M2 marker, CD206. The upper panels show IBA1 immunolabeling (white), while the matching lower panels show the merge for CD16/32 (magenta) and CD206 (green). **(A,E)** Microglia in the sham-vehicle mouse have small cell bodies, have thin processes, and are lightly labeled. **(B,F)** Microglia in the impact-vehicle mouse are larger and more intensely labeled. **(C,G)** Microglia in impact-ral5 mice largely resemble those in impact-vehicle mice. **(D,H)** Microglia in impact-ral10 mice are larger, rounder in shape, and more intensely labeled than microglia in the impact-vehicle mice. The green CD206 immunolabeling in **(H)** is more predominant than in **(F)**, reflecting the lower M1/M2 ratio for impact-ral10 mice. The scale bar in **(A)** applies to **(A–H)**. **(I–K)** Quantification of IBA1, CD16/32, and CD206 immunolabeling. Optic nerve area occupied by microglia, size, and roundedness of IBA1+ particles, expression of IBA1, CD16/32, and CD206, and M1/M2 ratio were all increased in impact-vehicle mice. Raloxifene at 5 mg/ml reduced CD16/32 and CD206 expression, M1/M2 ratio, optic nerve area occupied by microglia, and the size and roundedness of IBA1+ particles relative to impact-vehicle mice, but not IBA1 expression. Microglial area and size and roundedness of IBA1+ particles were greater for impact-ral10 mice than for impact-vehicle mice, IBA1 and CD206 were increased above impact-vehicle levels, but CD16/32 to a lesser extent, resulting in a lower M1/M2 ratio. Numbers of optic nerves analyzed: 10 sham-vehicle mice, 11 impact-vehicle mice, 12 impact-ral5 mice, and 15 impact-ral10 mice. Yellow asterisks indicate significant differences between the impact-ral10 mice and the sham-vehicle mice; *p < 0.05, **p < 0.01.

To further assess the effects of impact TBI and raloxifene treatment, we focused on the region of maximal optic nerve injury, i.e., beyond the extraocular muscle cone, and examined IBA1-immunolabeled microglia for expression of the M1 marker CD16/32 and the M2 marker CD206 (Figures 7A–H). We found increased expression of IBA1, CD16/32, and CD206, and increases in the M1/M2 ratio, the optic nerve area occupied by microglia, and the size and roundedness of IBA1+ particles after impact TBI (Figures 7I–K). Chi-square analysis showed that microglia in the impact-vehicle mice differed significantly from those in sham-vehicle mice for these seven traits (*p* = 0.037). Raloxifene at 5 mg/ml reduced CD16/32 and CD206 expression, the M1/M2 ratio, the optic nerve area occupied by microglia, and the size and roundedness of IBA1+ particles relative to impact-vehicle mice, but not IBA1 expression (Figures 7I–K). Mice treated with raloxifene at 10 mg/ml showed different changes in microglial characteristics than microglia in impact-vehicle or impact-ral5 mice that appear to have been protective, based on the nearly complete rescue of optic nerve axons (Figures 7I–K). Both IBA1 and CD206 were considerably elevated above impact-vehicle levels in impact-ral10 mice, but CD16/32 to a lesser extent, resulting in an M1/M2 ratio that was less than in impact-vehicle mice. The area occupied by microglia and the size and roundedness of IBA1+ particles were also increased compared to impact-vehicle mice, and more so relative to sham-vehicle mice. Chi-square analysis for the set of seven microglia traits showed that impact-ral10 mice were significantly different from sham-vehicle mice (*p* = 4.5 × 10^–7^) and impact-ral5 mice (*p* = 8.0 × 10^–4^), but not from impact-vehicle mice (*p* = 0.39). Interestingly, large, round microglia in the optic nerves of impact-ral10 mice tended to be clustered together more frequently and more closely associated with SMI-32+ axon bulbs than in either impact-vehicle or impact-ral5 mice. Some of these microglia appeared to be engulfing axon bulbs (Figures 6B,E), while other microglia were associated with small SMI-32+ profiles (Figures 6C,F) that corresponded to debris from fragmenting, degenerating axons (Del Mar et al., 2015).

#### Effect on Optic Tract Microglia

**FIGURE 8:**
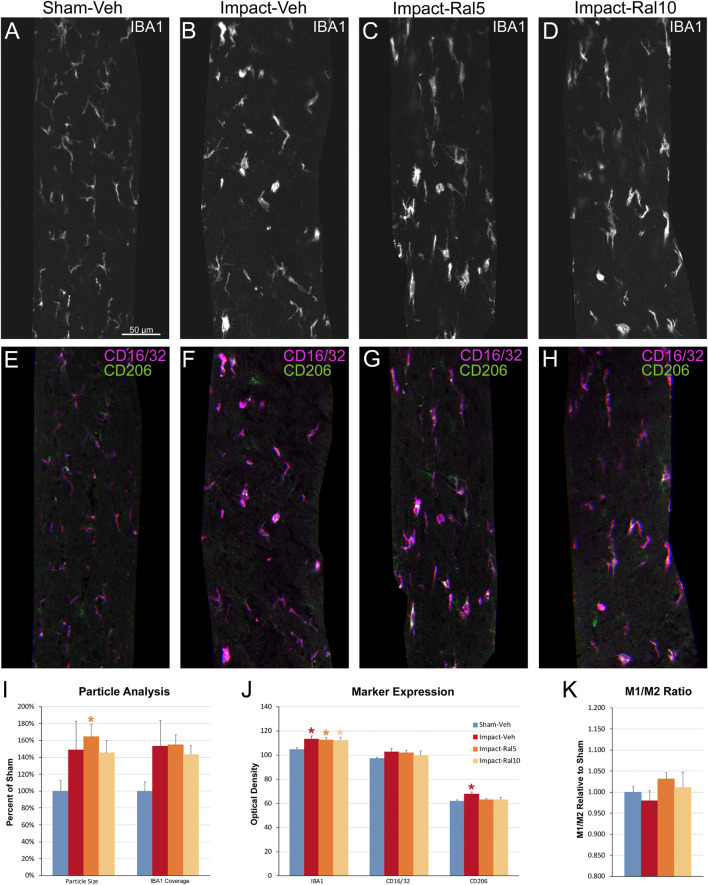
Microglia in the optic tract 3 days after impact TBI. **(A–H)** Confocal images of sections immunostained for IBA1 to visualize microglia, for the M1 marker, CD16/32, and for the M2 marker, CD206. The upper panels show IBA1 immunolabeling (white), while the matching lower panels show the merge for CD16/32 (magenta) and CD206 (green). **(A,E)** Microglia in the sham-vehicle mouse have small cell bodies, thin processes and are lightly labeled. **(B–D,F–H)** Microglia are larger and more intensely labeled in the impact mice with and without raloxifene treatment. The scale bar in **(A)** applies to **(A–H)**. **(I–K)** Quantification of IBA1, CD16/32, and CD206 immunolabeling. For impact-vehicle mice, optic tract area occupied by microglia and size of IBA1+ particles were increased, IBA1, CD16/32, and CD206 expression were all slightly increased, but CD206 expression showed a slightly larger increase than CD16/32, resulting in a slightly lower M1/M2 ratio than in sham mice. The two doses of raloxifene after impact TBI similarly were associated with an increased microglial area, size of IBA1+ particles, and IBA1 and CD16/32 expression. However, as CD206 expression in raloxifene-treated mice was slightly higher than in sham mice and less than in impact-vehicle mice, their M1/M2 ratios were higher. Numbers of optic tracts analyzed: 11 sham-vehicle mice, 15 impact-vehicle mice, 16 impact-ral5 mice, and 16 impact-ral10 mice. Red, orange, and yellow asterisks indicate significant differences between the impact-vehicle mice, impact-ral5 mice, and impact-ral10 mice, respectively, compared to the sham-vehicle mice; *p < 0.05.

We also examined the optic tract in these same mice using multiple immunofluorescent labeling for IBA1, CD16/32, and CD206 (Figures 8A–H). In the impact-vehicle mice, we found an increase in the size of IBA1+ particles, their coverage of the optic tract, and in the expression of IBA1, CD16/32, and CD206 (Figures 8I–K). The relative increase in CD206 expression was slightly greater than that for CD16/32, such that M1/M2 ratio for microglia in the impact-vehicle mice was less than that in sham mice, despite the clear evidence of microglial activation. Chi-square analysis showed that the impact-vehicle mice differed significantly from sham-vehicle mice for these six traits (*p* = 3.7 × 10^–7^). Interestingly, the relative increase in microglial coverage for the optic tract exceeded 50% (Figure 8I), and thus was more than twice the increase for the optic nerve (Figure 7I), whereas the increases in the intensity of IBA1, CD16/32, and CD206 immunolabeling for optic tract microglia (Figure 8J) were consistently less than for optic nerve microglia (Figure 7J). These outcomes seem to reflect the more focal nature of the damage to the optic nerve caused by impact TBI as compared to the optic tract. Raloxifene at both doses slightly decreased the intensity of IBA1 and CD206 immunolabeling for optic tract compared to impact-vehicle mice, with the higher dose also decreasing CD16/32 expression and microglial coverage of the optic tract (Figures 8I–K). Chi-square analysis showed that 5 mg/kg and 10 mg/kg raloxifene mice were significantly different from sham-vehicle mice (*p* = 8.6 × 10^–8^ and 1.6 × 10^–4^, respectively) but not from one another or from impact-vehicle mice.

#### Effect on Microglia in the Retina

We next combined immunolabeling for IBA1 with DAPI staining on sections of retina from these same mice. The impact-vehicle mice showed a small (∼30%) increase in total microglial abundance, and the percentage of microglia with cell bodies in outer retinal layers (outer plexiform layer and outer nuclear layer) was increased to ∼33% as compared to ∼23% in sham-vehicle mice. Increased abundance and redistribution of retinal microglia are known to be indicative of retinal damage (e.g., Santos et al., 2009). The magnitude of the changes after impact TBI was, however, considerably smaller than we found for the left eye after left-side focal cranial blast (Guley et al., 2019) and we did not observe obvious alterations of microglial morphology or increased IBA1 immunolabeling intensity. Retinas from the raloxifene-treated impact mice were similar to retinas from the impact-vehicle mice in the spatial distribution and abundance of microglia. Thus, IBA1 immunolabeling revealed only a small effect of impact TBI on retinal microglia and no obvious modulation with raloxifene. We did, however, observe effects of impact TBI and raloxifene on retinal microglia when we used molecular approaches to analyze a larger set of markers, as is described below.

#### qPCR and NanoString Analysis of Microglial Transcript Expression Levels

**FIGURE 9:**
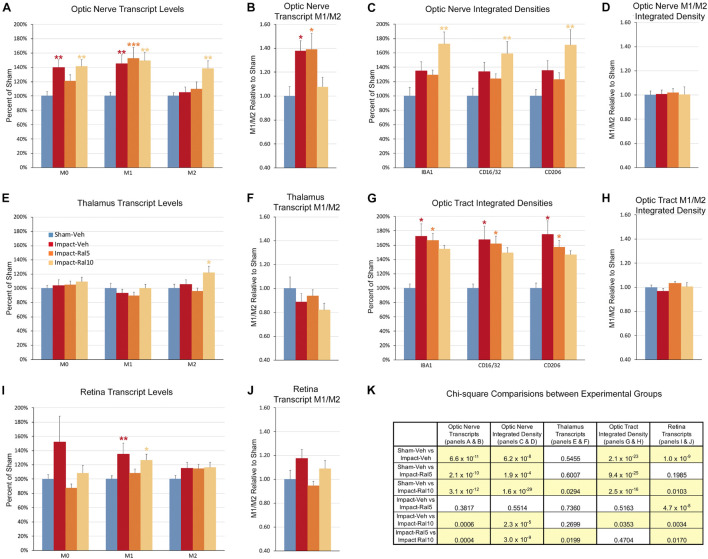
Expression of microglial markers 3 days after impact TBI. Levels of M0, M1, and M2 transcripts were determined by qPCR/NanoString for the optic nerve **(A,B)**, thalamus **(E,F)**, and retina **(I,J)**. Histograms show average levels relative to sham mice or the M1/M2 ratio relative to sham. Multiple immunofluorescence was performed for IBA1, CD16/32, and CD206 on sections of optic nerve **(C,D)** and optic tract **(G,H)** and quantified by determining the integrated optical density for each marker in the region of interest. Histograms show the total integrated density relative to sham or the M1/M2 ratio relative to sham. Red, orange, and yellow asterisks indicate significant differences between the impact-vehicle mice, impact-ral5 mice, and impact-ral10 mice, respectively, compared to the sham-vehicle mice; *p < 0.05, **p < 0.01, ***p < 0.001. **(K)** Chi-square analysis of mean M0 transcript levels or IBA1 integrated optical density, mean M1 transcript levels or CD16/32 integrated optical density, and mean M2 transcript levels or CD206 integrated optical density, and the M1/M2 ratio was used to compare across experimental groups to determine how these four endpoints (M0 marker, M1 marker, M2 marker, and M1/M2 ratio) differed as a set for each of the tissues examined. Comparisons yielding *p* < 0.05 are highlighted in yellow.

Both optic nerve (Figure 9A and Table 3) and retina (Figure 9I and Table 4) from the impact-vehicle mice exhibited increases in the overall expression of M0 transcripts, with IBA1, TGFβ, and Tmem119 most prominently increased in optic nerve and P2ry12, SPARC, and Tmem119 most prominently increased in retina. By contrast, the mean level of M0 transcripts in the thalamus was only slightly larger for impact-vehicle mice than for sham-vehicle mice (Figure 9E and Table 5). For optic nerve, impact produced a larger mean increase of M1 markers than of M2 markers, yielding a higher M1/M2 ratio (1.38) for impact-vehicle mice than for sham-vehicle mice (Figure 9B and Table 3). Several M1 markers (CD16, CD32, CD86, Tlr2, and TNFα) showed large increases with impact for optic nerve, while some M2 markers showed increased expression (CD206, Trem2, and Ym1), others decreased expression (Arg-1 and Fizz1), and the rest only small changes. The average levels of expression of both M1 and M2 transcripts for optic nerve were increased slightly more with 5 mg/ml raloxifene than with impact alone, resulting in a similar M1/M2 ratio as in impact-vehicle mice (1.39 vs. 1.38; Figures 9A,B). With 10 mg/ml raloxifene, the relative mean increase in M2 markers for optic nerve was considerably greater than in impact-vehicle mice (138.5% vs. 105.2%, respectively; *p* = 0.0047) and the relative mean increase in M1 markers was comparable (149.1% vs. 144.9%), yielding a lower M1/M2 ratio than in impact-vehicle mice (1.08 vs. 1.38) that trended toward statistical significance (*p* = 0.0513). Levels of Arg-1, CD36, CD206, Trem2, and Ym1 were notably increased with the 10 mg/ml dose of raloxifene, while levels of TNFα were markedly decreased (Table 3). Chi-square analysis comparing these four characteristics (i.e., M0, M1, and M2 transcript levels and M1/M2 ratio) for the optic nerve indicated that microglia in impact-ral10 mice differed significantly from microglia in both sham-vehicle (*p* = 3.1 × 10^–12^) and impact-vehicle mice (*p* = 0.0006), reflecting their biasing toward the M2 state (Figure 9K).

**TABLE 3:** Expression of microglial transcripts in optic nerve.

Target	Category	Method	Sham-Veh	Impact-Veh	Impact-Ral5	Impact-Ral10
IBA1	M0	qPCR	100.0%	181.7%	141.7%	194.2%
MERTK	M0	NanoString	100.0%	89.3%	91.7%	112.4%
P2ry12	M0	qPCR/Nano	100.0%	124.4%	93.5%	133.7%
SPARC	M0	NanoString	100.0%	134.8%	130.5%	138.6%
TGFβ	M0	qPCR	100.0%	159.6%	145.2%	141.3%
Tmem119	M0	qPCR/Nano	100.0%	148.0%	123.8%	128.0%

CD16	M1	NanoString	100.0%	157.1%	203.2%	192.6%
CD32	M1	qPCR/Nano	100.0%	228.4%	215.9%	306.9%
CD86	M1	NanoString	100.0%	170.6%	119.1%	214.2%
IFNγ	M1	qPCR/Nano	100.0%	85.2%	67.8%	84.0%
IL1β	M1	qPCR/Nano	100.0%	115.6%	109.3%	132.0%
IL6	M1	NanoString	100.0%	85.9%	91.0%	67.6%
IL12p40	M1	NanoString	100.0%	77.2%	75.3%	70.3%
iNOS	M1	qPCR/Nano	100.0%	99.3%	117.6%	110.5%
MCP-1	M1	NanoString	100.0%	82.1%	261.8%	79.9%
Tlr2	M1	NanoString	100.0%	250.5%	259.1%	303.4%
TNFα	M1	qPCR	100.0%	242.3%	156.8%	79.2%

Arg-1	M2	qPCR/Nano	100.0%	72.8%	54.2%	146.6%
CD36	M2	NanoString	100.0%	114.2%	64.5%	157.4%
CD206	M2	NanoString	100.0%	122.0%	130.6%	195.7%
Fizz1	M2	NanoString	100.0%	62.7%	81.6%	72.4%
IL4rα	M2	NanoString	100.0%	107.6%	112.2%	106.8%
IL10	M2	qPCR/Nano	100.0%	92.8%	115.4%	92.8%
IL13	M2	NanoString	100.0%	95.9%	93.6%	81.4%
Trem2	M2	qPCR/Nano	100.0%	146.8%	158.7%	173.6%
Ym1	M2	qPCR/Nano	100.0%	132.3%	176.8%	219.6%

All	M0	qPCR/Nano	100.0%	139.6%	121.0%	141.4%
All	M1	qPCR/Nano	100.0%	144.9%	152.4%	149.1%
All	M2	qPCR/Nano	100.0%	105.2%	109.7%	138.5%
All	M1/M2	qPCR/Nano	1.00	1.38	1.39	1.08

*Expression was assessed 3 days after impact TBI. Expression levels for transcripts examined by both qPCR and NanoString were averaged. Data are expressed as a percentage compared to sham-vehicle mice.*

**TABLE 4:** Expression of microglial transcripts in retina.

Target	Category	Method	Sham-Veh	Impact-Veh	Impact-Ral5	Impact-Ral10
IBA1	M0	qPCR	100.0%	74.7%	51.2%	124.5%
MERTK	M0	NanoString	100.0%	127.8%	97.6%	80.9%
P2ry12	M0	qPCR/Nano	100.0%	292.4%	92.7%	154.0%
SPARC	M0	NanoString	100.0%	162.7%	117.2%	107.6%
TGFβ	M0	qPCR	100.0%	109.9%	77.8%	112.3%
Tmem119	M0	qPCR/Nano	100.0%	145.5%	89.6%	71.5%

CD16	M1	NanoString	100.0%	260.6%	105.9%	123.3%
CD32	M1	qPCR/Nano	100.0%	192.3%	93.3%	116.3%
CD86	M1	NanoString	100.0%	148.8%	145.1%	135.5%
IFNγ	M1	qPCR/Nano	100.0%	100.6%	105.8%	112.6%
IL1β	M1	qPCR/Nano	100.0%	190.7%	133.4%	216.5%
IL6	M1	NanoString	100.0%	68.9%	86.2%	81.3%
IL12p40	M1	NanoString	100.0%	128.7%	157.7%	146.3%
iNOS	M1	qPCR/Nano	100.0%	133.6%	102.3%	136.3%
MCP-1	M1	NanoString	100.0%	73.9%	90.8%	91.6%
Tlr2	M1	NanoString	100.0%	142.9%	130.0%	105.5%
TNFα	M1	qPCR	100.0%	44.6%	42.9%	128.3%

Arg-1	M2	qPCR/Nano	100.0%	54.7%	58.3%	53.8%
CD36	M2	NanoString	100.0%	82.3%	70.9%	59.5%
CD206	M2	NanoString	100.0%	171.8%	146.2%	119.7%
Fizz1	M2	NanoString	100.0%	184.6%	151.8%	166.3%
IL4rα	M2	NanoString	100.0%	90.2%	104.8%	83.9%
IL10	M2	qPCR/Nano	100.0%	114.6%	133.2%	127.7%
IL13	M2	NanoString	100.0%	126.1%	156.7%	149.3%
Trem2	M2	qPCR/Nano	100.0%	84.7%	95.3%	100.9%
Ym1	M2	qPCR/Nano	100.0%	126.5%	114.3%	184.8%

All	M0	qPCR/Nano	100.0%	152.2%	87.7%	108.5%
All	M1	qPCR/Nano	100.0%	135.1%	108.5%	126.7%
All	M2	qPCR/Nano	100.0%	115.1%	114.6%	116.2%
All	M1/M2	qPCR/Nano	1.00	1.17	0.95	1.09

*Expression was assessed 3 days after impact TBI. Expression levels for transcripts examined by both qPCR and NanoString were averaged. Data are expressed as a percentage compared to sham-vehicle mice.*

**TABLE 5:** Expression of microglial transcripts in thalamus.

Target	Category	Method	Sham-Veh	Impact-Veh	Impact-Ral5	Impact-Ral10
IBA1	M0	qPCR	100.0%	109.1%	101.0%	89.2%
MERTK	M0	NanoString	100.0%	117.4%	141.6%	129.1%
P2ry12	M0	qPCR/Nano	100.0%	129.4%	139.8%	162.1%
SPARC	M0	NanoString	100.0%	72.7%	85.9%	104.8%
TGFβ	M0	qPCR	100.0%	102.6%	83.1%	88.5%
Tmem119	M0	qPCR/Nano	100.0%	91.5%	80.0%	83.2%

CD16	M1	NanoString	100.0%	88.1%	87.4%	109.8%
CD32	M1	qPCR/Nano	100.0%	116.5%	106.2%	134.7%
CD86	M1	NanoString	100.0%	124.3%	152.9%	186.4%
IFNγ	M1	qPCR/Nano	100.0%	111.8%	94.8%	87.0%
IL1β	M1	qPCR/Nano	100.0%	101.7%	78.8%	72.3%
IL6	M1	NanoString	100.0%	102.3%	109.4%	97.3%
IL12p40	M1	NanoString	100.0%	50.5%	57.2%	53.3%
iNOS	M1	qPCR/Nano	100.0%	91.2%	85.3%	103.5%
MCP-1	M1	NanoString	100.0%	66.1%	59.9%	49.8%
Tlr2	M1	NanoString	100.0%	86.1%	114.8%	140.8%
TNFα	M1	qPCR	100.0%	89.3%	42.0%	65.1%

Arg-1	M2	qPCR/Nano	100.0%	115.0%	82.0%	183.6%
CD36	M2	NanoString	100.0%	140.4%	123.2%	192.3%
CD206	M2	NanoString	100.0%	96.9%	105.6%	179.4%
Fizz1	M2	NanoString	100.0%	109.0%	85.8%	95.6%
IL4rα	M2	NanoString	100.0%	104.8%	103.9%	108.1%
IL10	M2	qPCR/Nano	100.0%	112.4%	115.5%	102.2%
IL13	M2	NanoString	100.0%	59.9%	67.8%	63.3%
Trem2	M2	qPCR/Nano	100.0%	92.9%	105.1%	92.9%
Ym1	M2	qPCR/Nano	100.0%	116.8%	74.5%	80.6%

All	M0	qPCR/Nano	100.0%	103.8%	105.2%	109.5%
All	M1	qPCR/Nano	100.0%	93.4%	89.9%	100.0%
All	M2	qPCR/Nano	100.0%	105.3%	96.0%	122.0%
All	M1/M2	qPCR/Nano	1.00	0.89	0.94	0.82

*Expression was assessed 3 days after impact TBI. Expression levels for transcripts examined by both qPCR and NanoString were averaged. Data are expressed as a percentage compared to sham-vehicle mice.*

In the case of retina, impact also produced a larger mean increase of M1 markers than of M2 markers, yielding a higher M1/M2 ratio (1.17) for impact-vehicle mice than for sham-vehicle mice (Figures 9I,J). Among M1 transcripts, CD16, CD32, CD86, IL1β, and Tlr2 showed the greatest increases with impact, as did CD206 and Fizz1 among the M2 transcripts (Table 4). The lower dose of raloxifene significantly reduced M1 transcripts overall (particularly CD16, CD32, and IL1β) relative to impact-vehicle mice (*p* = 0.0376), but produced little change in M2 transcripts, yielding an M1/M2 ratio less than that for impact-vehicle mice (0.95 vs. 1.17) that trended toward statistical significance (*p* = 0.0713). With the higher raloxifene dose, mean M1 expression remained elevated relative to sham-vehicle mice (with smaller decreases in CD16 and CD32 levels than produced by the lower dose and an increase in IL1β levels relative to impact alone) and was only slightly reduced relative to impact-vehicle mice. Overall M2 transcript levels were similar to those in impact-vehicle mice (except for an increase in Ym1 levels), resulting in an M1/M2 ratio (1.09) slightly less than for impact-vehicle mice (1.17) but higher than for sham mice (1.00) and for impact mice with the lower raloxifene dose (0.95) (Figures 9I,J and Table 4). Chi-square analysis for the retina showed that microglia in impact-ral10 mice differed significantly from sham-vehicle mice (*p* = 0.0103) and impact-vehicle mice (*p* = 0.0034) for the set of microglial expression traits examined, whereas microglia in impact-ral5 mice differed significantly from impact-vehicle mice (*p* = 4.7 × 10^–8^) but not from sham-vehicle mice (*p* = 0.1985) (Figure 9K). Thus, with the lower dose of raloxifene, microglia showed lower expression of markers characteristic of the pro-inflammatory M1 state.

In the case of thalamus (which contains optic tract, as well as several retinorecipient nuclei), microglia in impact-vehicle mice exhibited a slight decrease in M1 markers and a slight increase in M2 markers, yielding an M1/M2 ratio (0.89) slightly less than in sham mice (1.00) (Figures 9E,F and Table 5). The lower raloxifene dose slightly reduced both M1 and M2 markers below that in impact-vehicle mice and sham-vehicle mice (M2 markers more so), resulting in an M1/M2 ratio (0.94) similar to that in impact-vehicle mice and less than in sham-vehicle mice. The higher raloxifene dose increased M2 markers (*p* = 0.0141 relative to sham) (notably Arg-1, CD36, and CD206) more than it increased M1 markers, yielding an M1/M2 ratio (0.82) lower than for sham, impact alone, or impact mice treated with the lower raloxifene dose. Chi-square analysis indicated the only significant differences between groups for microglia in the thalamus were between impact-ral10 mice and sham-vehicle mice, and between impact-ral10 mice and impact-ral5 mice (Figure 9K).

#### Comparison Between Results Using Immunohistochemical and Molecular Analyses

We then compared the results from the molecular analysis of transcript levels with the immunolabeling assessments of relative levels of protein expression for the optic nerve and optic tract (Figure 9). For this analysis, to reflect the signals for the entire nerve/tract, we multiplied the average optical density for each immunolabeled marker by the percent microglial coverage for that particular optic nerve/tract, to obtain values of integrated optical densities. For the optic nerve, total IBA1, CD16/32, and CD206 immunolabeling signal levels were increased to similar extents above sham in the impact-vehicle mice (Figure 9C), but the molecular analysis revealed a larger net increase for the M1 transcripts than for the M2 transcripts (Figure 9A). The 5 mg/ml raloxifene dose reduced M0 transcript levels, but not M1 and M2 transcript levels, below the levels in impact-vehicle mice (Figure 9A), whereas immunolabeling showed small reductions for the M0 marker (IBA1), the M1 marker (CD16/32), and the M2 marker (CD206) (Figure 9C). By contrast, both approaches indicated that the 10 mg/ml raloxifene dose increased levels of the M2 markers above that in impact-vehicle mice. Importantly, chi-square analysis comparing the four traits for both the immunolabeling and molecular analysis for optic nerve microglia indicated that impact-ral10 mice were significantly different from sham-vehicle mice and impact-vehicle mice, whereas impact-ral5 mice were significantly different from sham-vehicle mice but not from impact-vehicle mice (Figure 9K). Taken together, the two analyses indicate that the higher raloxifene dose had a greater effect on optic nerve microglia than the lower dose. In the case of optic tract, impact-vehicle mice showed large increases in the integrated optical densities for IBA1, CD16/32, and CD206 immunolabeling (Figure 9G). Optic tract microglia in impact-ral5 mice exhibited smaller increases compared to sham, and microglia in impact-ral10 mice even smaller increases for the IBA1, CD16/32, and CD206 integrated optical densities (Figure 9G). Chi-square analysis comparing the immunolabeling and M1/M2 ratios indicated that both impact-ral5 mice and impact-ral10 mice differed significantly from sham-vehicle mice, and impact-ral10 mice, but not impact-ral5 mice, also differed significantly from impact-vehicle mice (Figure 9K). By contrast, with the exception of a significant elevation of M2 transcripts in impact-ral10 mice, the molecular analysis for the thalamus showed only small changes in transcript levels (Figure 9E). The discordance between our finding that the levels of M0, M1, and M2 transcripts for thalamus showed little or no increase in impact-vehicle mice and our finding that the intensities of IBA1, CD16/32, and CD206 immunolabeling for optic tract were substantially elevated in impact-vehicle mice is likely to reflect the fact the optic tract constitutes ∼1% of the volume of the tissue used to extract RNA from the thalamus. Thus, optic tract microglia would account for only a small percentage of the transcripts analyzed, and the levels of microglial transcripts for thalamus would not necessarily mirror the changes in expression for optic tract microglia.

## Discussion

In the work reported here, we found that raloxifene rescues visual deficits and associated pathologies that otherwise ensue from a single impact to the dorsal surface of the head. Below we describe these deficits, pathologies, and their rescue by raloxifene, and compare our results with previous studies. We then discuss the mechanism of raloxifene benefit and its potential use as a therapy for patients who experience TBI.

### Visual Deficits and Pathology After Impact Traumatic Brain Injury as Compared to Focal Cranial Blast Traumatic Brain Injury

There are several important similarities and differences to note between the dorsal cranial impact approach to creating mild TBI we used here and the high-pressure air blast model we used in our previous studies (Guley et al., 2016, 2019; Honig et al., 2019). The most obvious difference is that dorsal impact affects the two eyes and nerves equally whereas focal cranial blast delivered to the left side more prominently affects the left eye and optic nerve than the right eye and nerve. Because of this, for the current impact TBI studies, we pooled the data for both sides, and in the discussion below, we compare these pooled results to the data for left eye and optic nerve after focal cranial blast TBI. Less obvious is the fact that, when impact and blast impinge upon the skull, they both generate intracranial pressure gradients. These pressure gradients in turn generate biomechanical forces (e.g., shear, strain, and stretch) to which axons are highly sensitive. The precise magnitude, direction, and duration of these forces vary between regions of the brain and over time, albeit in ways that are not well understood, and may then differ in the pathological sequelae they initiate (Risling et al., 2019; Smith et al., 2021). In accord with this, we found similarities but also several important differences in the outcomes from the two types of TBI.

#### Contrast Sensitivity and Visual Acuity

Dorsal cranial impact and left side focal cranial blast both yielded bilateral deficits in contrast sensitivity, and a lesser if any deficit in visual acuity. The ∼10% reduction in contrast sensitivity for the two eyes after dorsal cranial impact was smaller than the ∼17% reduction for the left eye after cranial blast (Honig et al., 2019). Interestingly, the contrast sensitivity threshold was significantly correlated with optic nerve axon loss after focal cranial blast, but not after impact TBI, suggesting that the deficit following focal blast, but not that following dorsal impact, stems in large part from the loss of optic nerve axons (discussed further below). Raloxifene rescued the contrast sensitivity deficits produced by both impact and blast TBI, indicating that, nonetheless, the underlying pathologies involved microglial activation in both cases.

#### Scotopic Flash-Evoked Electroretinogram

Impact TBI decreased the peak amplitude of the B-wave of the scotopic ERG, which is rod bipolar cell-driven, but not the A-wave, which is rod photoreceptor-driven, whereas blast TBI decreased the peak amplitude of both. Neither the impact nor the blast directly impinged upon the eyes, but the intracranial pressure waves they each generated would have subsequently been transmitted from the brain forward through the orbit and then across the retina. Yet, impact TBI seemed to be less deleterious for rod photoreceptors than blast TBI. In accord with this, we found only a small increase (∼30%) in the abundance of microglia in the retina 3 days after impact, as compared to the tripling we previously reported following blast TBI (Guley et al., 2019). The B-wave deficit after impact TBI was rescued by the lower raloxifene dose, but not by the higher dose, suggesting that raloxifene benefit for rod bipolar cell injury was diminished with the higher dose. Consistent with this outcome, our molecular studies indicated the lower dose more effectively biased retinal microglia away from pro-inflammatory M1 state, as we will discuss later.

#### Light Aversion

Impact TBI produced an increase in light aversion, which was rescued by the higher but not the lower dose of raloxifene. These results are very similar to our previous findings for focal cranial blast TBI (Honig et al., 2019). There we attributed the increased light aversion to ipRGCs, which constitute 2–3% of retinal ganglion cells (RGCs) (Hughes et al., 2013) and have been implicated in light aversion (Matynia et al., 2012; Collison et al., 2015), increasing their expression of melanopsin. Here preliminary examination showed that impact TBI did not similarly enhance ipRGC melanopsin expression. It is possible that the subset of nociceptive trigeminal neurons that are localized to the ophthalmic branch of the trigeminal nerve and innervate the cornea (Matynia et al., 2016) instead upregulate their expression of melanopsin. Alternatively, melanopsin-expressing trigeminal afferent fibers in the conjunctiva, cornea, sclera, and/or uvea may transmit pain signals that contribute to photophobia (Matynia et al., 2015), and/or the pain-modulating neurons of the ventromedial raphe nucleus of the medulla may increase their pro-pain responses to their visual input (Martenson et al., 2016), thereby resulting in the enhanced light aversion we observed after impact TBI.

#### Pupil Light Reflex

Impact TBI caused an increase in resting pupil size, presumably due to damage to the circuit mediating pupil constriction, either centrally or *via* the disruption of preganglionic or postganglionic ciliary ganglion axons innervating iris sphincter muscles. The percent constriction to red or blue light was, however, no different than for sham, indicating that dynamic responses to light were not notably impaired, as we had also found after blast TBI (Honig et al., 2019). Interestingly, raloxifene at 10 mg/ml, but not at 5 mg/ml, rescued the diminished pupil constriction to red and blue light caused by impact TBI, and similarly rescued the enhancement in light aversion.

#### Optic Nerve

Impact TBI resulted in a nearly 22% loss of optic nerve axons, which was partly rescued by the low dose of raloxifene and completely by the high dose. The downward displacement of skull and brain during dorsal impact is likely to decrease the height of the bony optic nerve canal and thereby compress the optic nerve (Evanson et al., 2018). Indeed, compression of the optic nerve within the bony canal appears to have been the major cause of retinal axon injury, based on the prominence of swollen axon bulbs and activated microglia in this region a few days after the impact. Surprisingly, focal cranial blast TBI produced considerably less optic nerve axon loss (∼10%) and that loss was significantly correlated with the contrast sensitivity deficit. By contrast, RGC axon loss and contrast sensitivity are not correlated after impact TBI, suggesting that retinal injury, optic nerve dysfunction not manifesting as axon loss, and/or injury to central visual areas and pathways contribute to the loss of contrast sensitivity deficit produced by impact TBI. That dorsal impact injures the retina is supported by B-wave deficit and by the increase in abundance and spatial distribution of retinal microglia we observed, as well as by the thinning of the retina after repeat impact reported by the Crawford laboratory (Tzekov et al., 2014). Damage to central visual areas and/or white matter tracts by dorsal impact also seems likely, based on the axonal injury and microglial activation we found in the optic tract and by that observed in the corpus callosum (Mouzon et al., 2012, 2014).

### Visual Deficits in Other Models of Blunt Impact Injury Traumatic Brain Injury

Previous studies in mice using impact and impact-acceleration approaches have shown that forces delivered to the dorsum of the head typically injure axons of retinal origin. Damaged and/or degenerating axons are commonly observed in the optic nerve and optic tract, associated with the presence of activated microglia and the increased expression of astrocytic glial fibrillary acidic protein (GFAP) (Tzekov et al., 2014, 2016a, b; Bolton-Hall et al., 2016; Xu et al., 2016; Evanson et al., 2018; Das et al., 2019; Welsbie et al., 2019; Desai et al., 2020). Some reports have shown that pathological changes extend proximally to the cell bodies of origin and distally to synaptic terminals. For example, RGC loss and activated retinal microglia were observed in one study (Xu et al., 2016), and in another study, degenerating axons and reactive astrocytes were observed in two retinal target areas, specifically the dorsal lateral geniculate nucleus and the superior colliculus (Evanson et al., 2018). Interestingly, the suprachiasmatic nucleus, where ipRGCs terminate, did not show similar signs of injury, indicating that ipRGCs may be more resistant to axonal injury than other RGCs.

To the best of our knowledge, only a few studies of impact TBI in mice have assessed visual function. Of these, mice subjected to impact followed by rotational acceleration were found to make more errors on a visual cliff task and have smaller visual evoked field potentials when the injury was repeated on three successive days, but not after a single injury (Desai et al., 2020). In another study, mice that received five repeated impacts, spaced 2 days apart, using the same impact parameters as in our studies, showed a significant reduction in the amplitude of the photopic negative response, consistent with the ∼67% loss of RGCs they observed, but not in the amplitudes of the A- and B-waves, despite the thinning of the retinal inner and outer nuclear layers (Tzekov et al., 2014). As might be expected, immunostaining for GFAP and for IBA1 in the optic tract/nerve showed that both were still elevated many weeks after the initial injury in both studies.

### Microglial Modulation by Impact Traumatic Brain Injury and Raloxifene

As noted above, microglia become activated following TBI, rapidly upregulate their expression of CB2, and serve as the target of our CB2 inverse agonist treatment strategy. CB2 inverse agonists act by stabilizing CB2 receptors, which are otherwise constitutively active, in an inactive state, which has the ultimate overall effect of shifting microglia away from the pro-inflammatory M1 state and toward the protective M2 state (Lunn et al., 2006, 2008; Lawrence and Natoli, 2011; Franco and Fernández-Suárez, 2015). We previously showed that the CB2 inverse agonist SMM-189 modulates microglia in this manner *in vitro* (Presley et al., 2015; Reiner et al., 2015) and *in vivo* (Bu et al., 2016; Guley et al., 2019), and rescues various sensory, motor, and emotional deficits and the accompanying axon and neuron loss that mice otherwise exhibit after focal cranial blast TBI (Reiner et al., 2015; Bu et al., 2016; Guley et al., 2019). However, as neither SMM-189 nor the commercially available CB2 inverse agonist SR144528 have undergone any human testing^2^ (accessed April 29, 2021), their use in people is at least a decade off. By contrast, raloxifene, which also possesses CB2 inverse agonism (Kumar and Song, 2013; Honig et al., 2019) is already FDA-approved as a selective estrogen receptor modulator.

In previous work, we demonstrated that raloxifene, like SMM-189, greatly mitigates visual system injury and pathology after focal cranial blast TBI (Honig et al., 2019). We also showed that microglia in the retina, optic nerve, and optic tract are biased toward the pro-inflammatory M1 state shortly after the injury (Guley et al., 2019; Honig et al., 2019) and that raloxifene benefit is associated with shifting microglia away from the M1 state toward the M2 state (Honig et al., 2019). Note that we fully realize that microglia comprise a multidimensional spectrum of phenotypes (Hanisch, 2013; Franco and Fernández-Suárez, 2015; Bennett et al., 2016; Jha et al., 2016; Morganti et al., 2016; Fernández-Arjona et al., 2017; Honig et al., 2020; Mesquida-Veny et al., 2021). With this in mind, in the current work, we expanded upon the use of immunolabeling for IBA1, CD16/32, and CD206 to characterize individual microglia (Guley et al., 2019; Honig et al., 2019) by also employing qPCR/NanoString technology to assess the expression of a larger number of microglial markers and to provide a better overview of the overall inflammatory/protective milieu. Thus, although microglia are not limited to two distinct polarization states, the relative bias toward M1 or M2 markers, as encapsulated in the M1/M2 marker ratio, provides a useful means for describing microglial modulation and its potential role in exacerbating or mitigating TBI outcome.

#### Optic Nerve

In the present study, we found that microglia in the optic nerve were biased toward the M1 phenotype after impact TBI. The higher dose of raloxifene, but not the lower dose, reduced the M1/M2 ratio relative to impact-vehicle mice by increasing the overall expression of M2 transcripts. Among the M1 transcripts showing large increases with impact, CD16 and CD32 are membrane proteins that bind the Fc portion of immunoglobulin gamma antibodies, CD86 is a co-stimulatory membrane receptor involved in IL-2 production, and Tlr2 is a pattern recognition receptor that binds DAMPs (as well as pathogens) and participates in triggering subsequent pro-inflammatory responses (Sordillo et al., 2016; Jurga et al., 2020). Interestingly, TNFα, a major pro-inflammatory cytokine that initiates diverse downstream signaling events, including cell death, and promotes the transcription of other pro-inflammatory genes (Rodríguez-Gómez et al., 2020), was both markedly increased with impact and markedly decreased by 10 mg/ml raloxifene. The higher dose of raloxifene also produced notable increases in several M2 transcripts relative to impact-vehicle mice, specifically Arg-1, CD36, CD206, Trem2, and Ym1. Arg-1 is an enzyme that competes with iNOS for arginine; with an increase in Arg-1 transcripts, the levels of nitric oxide, which is cytotoxic, would be diminished (Fouda et al., 2020). Trem2 is a membrane receptor that binds a wide variety of ligands and is best known for its protective role in Alzheimer’s disease. Trem2 binding to extracellular ligand and adaptor proteins in microglia stimulates several downstream signaling pathways, ultimately boosting cellular metabolism, and allowing microglia to serve protective roles (Ulland and Colonna, 2018; Qin et al., 2021). Ym1 is thought to sense and regulate inflammatory responses, acts to maintain the extracellular matrix, and has been shown to play an important role in mitigating neural injury after stroke (Im et al., 2020; Zhao et al., 2020). CD36 and CD206 are both involved in phagocytosis and will be discussed further below.

The immunolabeling results similarly revealed a significant M2 marker (CD206) elevation with the higher dose. In some sections from mice that had received impact, we observed microglia possessing especially rounded cell bodies. The rounded microglia tended to be localized to the site of maximal axonal injury (corresponding to the region within and just beyond the bony optic canal) and were more abundant in impact-ral10 mice than in impact-ral5 mice or impact-vehicle mice. In some cases, the rounded microglia appeared to be engulfing swollen axon bulbs, and in other cases, they were intermingled with small SMI-32+ profiles that resembled debris from damaged axons (Del Mar et al., 2015). Together, these observations suggest that the rounded optic nerve microglia may have been in the process of clearing axonal debris by phagocytosis. The removal of cellular debris is significant because that material would otherwise accumulate and enhance deleterious pro-inflammatory responses (Márquez-Ropero et al., 2020). In this regard, it is important to note that recent studies have revealed that varicose swellings form along some intact axons when nerves are damaged (Weber et al., 2019; Gu, 2021). Axons with such varicosities are still viable and, as such, salvageable, in contrast to axons with terminal bulbs, which are indicative of a disconnection, with the distal part of the axon ultimately undergoing Wallerian degeneration. It seems likely, based on the considerable optic nerve axon loss in impact-vehicle mice, that many of the axon swellings initially formed progress to being terminal bulbs, with the disconnected distal nerve later degenerating. By contrast, very few optic nerve axons were lost in impact-ral10 mice. In these mice, the removal of terminal bulbs and degenerating distal axon fragments by phagocytosis may have reduced the pro-inflammatory environment, allowing the less-damaged axons to be rescued. The additional trophic actions of the M2-biased microglia may have also facilitated such rescue, so that optic nerve axon loss was statistically undetectable in the impact-ral10 mice. On the other hand, microglia show less of a shift toward the M2 phenotype in the impact-ral5 mice, and so, fewer of the less-damaged optic nerve axons may be rescued. Interestingly, we found a significant negative correlation between the aspect ratio of the IBA1+ profiles and CD206 expression levels across all experimental groups (*p* = 0.0387), but no correlation with the expression of IBA1 or CD16/32, or the M1/M2 ratio. That is, rounder microglia (those with lower aspect ratios), which were more characteristic of impact-ral10 mice, tended to express higher levels of CD206. CD206 is a mannose receptor and acts in phagocytosis (Franco and Fernández-Suárez, 2015). Treatment with 10 mg/ml raloxifene similarly increased the expression of the M2 marker CD36, a scavenger protein that helps to clear myelin debris (Grajchen et al., 2020).

#### Optic Tract and Thalamus

For the optic tract, immunolabeling showed that impact produced an ∼3% decrease in the M1/M2 ratio due to a larger increase in CD206 expression than in CD16/32 expression (∼175% vs. ∼168% above sham, respectively), and the molecular analysis showed an ∼7% decrease in the M1/M2 ratio for the thalamus due to ∼5% decrease in M1 transcripts and ∼2% increase in M2 transcripts. Treatment with raloxifene resulted in relatively small changes in the levels of M1 and M2 transcripts in thalamus, with the exception of an elevation in M2 transcripts in impact-ral10 mice. By contrast, the integrated optical densities for both CD16/32 and CD206 in optic tract were slightly decreased in impact mice treated with raloxifene relative to impact alone, and somewhat more so for the higher dose than for the lower dose. These discrepancies between the immunolabeling and the molecular results can at least partly be explained by the fact that the optic tract constituted only ∼1% of the volume of the tissue we used for extracting RNA from the thalamus. Optic tract microglia would thus have made a relatively small contribution to the total thalamic transcriptome. Accordingly, the immunolabeling results for the optic tract likely provide a better indication of how microglia respond to the axonal injury produced by impact TBI. Comparing the optic tract to the optic nerve with regard to M2 markers, M2 expression in impact-ral10 mice was reduced relative to impact-vehicle mice for optic tract (as indicated by immunolabeling), whereas M2 expression was elevated for optic nerve (as indicated by both immunolabeling and molecular analysis). An explanation for this difference may lie in the relatively greater axon injury produced by the impact in optic nerve than in optic tract. As raloxifene specifically targets microglia by binding to CB2 receptors, the CB2 upregulation that accompanies microglial activation would be required for raloxifene treatment to be effective. The lesser microglial activation in the optic tract may be associated with less of an increase in CB2 expression, and accordingly raloxifene may be less effective in modulating microglia in optic tract toward an M2 state, compared to microglia in optic nerve.

#### Retina

For the retina, the molecular analysis showed that microglia in the impact-vehicle mice were biased toward the M1 phenotype, with CD16, CD32, CD86, IL1β, and Tlr2 showing the greatest increases. The lower dose of raloxifene reduced M1 transcripts (particularly CD16, CD32, and IL1β) relative to impact-vehicle mice, but produced only small changes in M2 transcripts. The higher dose had little effect on the mean expression level for either M1 or M2 transcripts. The lower dose was thus more effective than the higher dose in biasing microglia away from the pro-inflammatory M1 state after impact TBI. It is uncertain why this may be, but the decrease in M1 expression with the lower dose may have played a role in its greater efficacy in rescuing the B-wave of the ERG. IL1β is a potent pro-inflammatory cytokine (Sordillo et al., 2016) and is commonly upregulated after TBI (e.g., Loane and Kumar, 2016; Morganti et al., 2016; Madathil et al., 2018). In the retina, IL1β has been shown to promote retinal neuroinflammation and drive neurodegeneration, for example, in photoreceptor degeneration, diabetic retinopathy, and age-related macular degeneration (Wooff et al., 2019; Charles-Messance et al., 2020). The interpretation that decreased IL1β expression by the lower raloxifene dose may have been beneficial for retina after impact TBI is consistent with recent evidence that pharmacological blockade of IL1 signaling mitigates retinal injury after repeated blast TBI (Evans et al., 2020).

#### Microglia Modulation and Treatment Duration

Given that microglia remain activated beyond the 3 days we used here to assess microglial states, it is likely that continuing the raloxifene treatment for a longer period would be even more effective in decreasing M1 markers and increasing M2 markers. In this regard, it is important to note that the mice used for functional testing and axon counts received raloxifene daily for 2 weeks. Furthermore, although the reasons for the variation in rescue for the two doses we tested are not entirely certain, they do not appear to reflect drug toxicity, as raloxifene-treated mice did not weigh less than sham-vehicle mice at the time of sacrifice in the present study or after focal cranial blast TBI (Honig et al., 2019).

### Considerations for Human Use

In principle, raloxifene benefit in ameliorating visual system injury after TBI could stem from its estrogenic effects (Frick et al., 2015; Habib and Beyer, 2015), specifically its action as an agonist at β-type estrogen receptors (ER-β), or as an antagonist at α-type estrogen receptors (ER-α). This, however, does not seem to be true, as demonstrated by our previous experiments showing that the contrast sensitivity deficit resulting from focal cranial air blast was not rescued by an ER-β agonist in combination with an ER-α antagonist, nor did an ER-β antagonist block the improvement in contrast sensitivity produced by raloxifene (Honig et al., 2019). Moreover, the possibility that raloxifene’s antagonism at ER-α could be anti-inflammatory is inconsistent with the recent finding that 17β-estradiol exerts anti-inflammatory effects by acting as an agonist at ER-α receptors in adult male mice after an impact producing more severe injury (Wang et al., 2021).

Two additional considerations support the view that raloxifene benefit in TBI is mediated by CB2 receptors. First, we previously showed that raloxifene and the CB2 inverse agonist SR144528 showed similar benefit for the contrast sensitivity deficit after focal cranial air blast (Honig et al., 2019). Second, as discussed in the previous section, here we found that raloxifene action on microglia was more pronounced in optic nerve where the trauma was greater, likely producing a larger elevation in CB2 receptor expression, than in the optic tract where the extent of microglial activation and, hence, of CB2 upregulation were likely to have been minimal.

Raloxifene was approved by the FDA to treat postmenopausal osteoporosis in 1999 and for reducing the risk of invasive breast cancer in postmenopausal women in 2007. Importantly, as we previously discussed in more detail (Honig et al., 2019), raloxifene has no evident adverse hormonal side effects and is also safe for use in men. We tested doses that are 5- to 10-fold higher than the 60 mg/day prescribed for osteoporosis and cancer in humans, to ensure that an adequate concentration of drug entered the mouse brain, and found benefit for visual deficits and visual system injury after impact TBI in the current studies and after focal cranial blast TBI in our previous work (Honig et al., 2019). Doses this high (600 mg/day) are, however, known to be safe in humans (Draper et al., 1996). Our unpublished results also indicate that raloxifene reduces the increased fearfulness and depression mice exhibit after focal cranial blast TBI (Heldt et al., 2014) as did SMM-189 (Reiner et al., 2015; Bu et al., 2016). Thus, individuals who have suffered a concussion and might otherwise experience persisting, psychologically debilitating symptoms (van der Naalt et al., 2017; Stein et al., 2019) may greatly benefit if raloxifene treatment is initiated within a few days of the trauma.

Although we used male mice for our studies, it would also be useful to evaluate raloxifene efficacy in female mice. The impactor approach we employed to produce mild TBI has been used to study male mice exclusively, but outcomes using other TBI approaches that produce more severe injuries have not shown consistent sex differences (see reviews by Spani et al., 2018; Gupte et al., 2019). Similarly, there is no clear evidence for consistent sex differences in microglial abundance or behavior, CB2 expression levels, or cytokine production in healthy adult rodents (Crain et al., 2013; Marco et al., 2013; Xing et al., 2014; Lyu et al., 2021), and even whether there are differences in TBI outcomes for men and women has not been clearly established (Spani et al., 2018; Gupte et al., 2019). Thus, while current evidence gives no reason to expect significant differences in raloxifene efficacy for TBI between males and females, it would nonetheless be valuable to examine this directly.

Another important consideration is when treatment should be initiated and how long it should continue. Consistent with the early pro-inflammatory behavior of microglia after focal cranial blast TBI, we found that raloxifene therapy should start no later than day 3 for maximum benefit (Honig et al., 2019). However, we do not know if 2 weeks of treatment is needed and further work is required to optimize dosage. Taken together, our findings that raloxifene rescues functional deficits and pathological changes following both focal cranial blast and impact TBI support its further consideration. Phase 2 clinical trials could start at any time, and if found effective, raloxifene could be repurposed as a therapy for mild TBI relatively quickly.

## Data Availability Statement

The original contributions presented in the study are included in the article/supplementary material, further inquiries can be directed to the corresponding author/s.

## Ethics Statement

The animal study was reviewed and approved by Department of Defense (DOD) and The University of Tennessee Health Science Center (UTHSC) Animal Care and Use Committees.

## Author Contributions

MH and AR planned the studies, acquired the confocal images, analyzed the data, and wrote the manuscript. ND and DH performed the TBI and behavioral studies. DO’N and JD analyzed the data. RC performed the behavioral and molecular studies. CL performed the histological studies. AP performed the TBI, molecular studies, and analyzed the results. BM formulated drug. All authors read and approved the manuscript.

## Conflict of Interest

The authors declare that the research was conducted in the absence of any commercial or financial relationships that could be construed as a potential conflict of interest.

## Publisher’s Note

All claims expressed in this article are solely those of the authors and do not necessarily represent those of their affiliated organizations, or those of the publisher, the editors and the reviewers. Any product that may be evaluated in this article, or claim that may be made by its manufacturer, is not guaranteed or endorsed by the publisher.

## Footnotes

1
www.cerebralmechanics.com

2
ClinicalTrials.gov
